# Chromatin architecture at susceptible gene loci in cerebellar Purkinje cells characterizes DNA damage–induced neurodegeneration

**DOI:** 10.1126/sciadv.abg6363

**Published:** 2021-12-15

**Authors:** Young Don Kwak, Timothy I. Shaw, Susanna M. Downing, Ambika Tewari, Aditi, Hongjian Jin, Yang Li, Lavinia C. Dumitrache, Sachin Katyal, Kamran Khodakhah, Helen R. Russell, Peter J. McKinnon

**Affiliations:** 1 Departments of Genetics and Cell Molecular Biology, Center for Pediatric Neurological Disease Research, St. Jude Pediatric Translational Neuroscience Initiative, Memphis, TN 38105, USA.; 2 Moffitt Cancer Center, Tampa, FL 33612, USA.; 3 Dominick P. Purpura Department of Neuroscience, Albert Einstein College of Medicine, Bronx, NY 10461, USA.; 4 Center for Applied Bioinformatics, St. Jude Children’s Research Hospital, Memphis, TN 38105, USA.; 5 CancerCare Manitoba Research Institute, CancerCare Manitoba and Department of Pharmacology and Therapeutics, University of Manitoba, Winnipeg, MB R3E OV9, Canada.; 6 St. Jude Graduate School of Biomedical Sciences, Memphis, TN 38105, USA.

## Abstract

The pathogenesis of inherited genome instability neurodegenerative syndromes remains largely unknown. Here, we report new disease-relevant murine models of genome instability–driven neurodegeneration involving disabled ATM and APTX that develop debilitating ataxia. We show that neurodegeneration and ataxia result from transcriptional interference in the cerebellum via aberrant messenger RNA splicing. Unexpectedly, these splicing defects were restricted to only Purkinje cells, disrupting the expression of critical homeostatic regulators including *ITPR1*, *GRID2*, and *CA8*. Abundant genotoxic R loops were also found at these Purkinje cell gene loci, further exacerbating DNA damage and transcriptional disruption. Using ATAC-seq to profile global chromatin accessibility in the cerebellum, we found a notably unique chromatin conformation specifically in Purkinje chromatin at the affected gene loci, thereby promoting susceptibility to DNA damage. These data reveal the pathogenic basis of DNA damage in the nervous system and suggest chromatin conformation as a feature in directing genome instability–associated neuropathology.

## Teaser

Chromatin architecture of critical Purkinje cell genes predicts cerebellar degeneration after genome damage.

## INTRODUCTION

The nervous system is susceptible to DNA damage, and inherited human syndromes characterized by DNA repair defects feature profound neuropathology including neurodegeneration, microcephaly, or brain tumors (*1*, *2*). However, while the need to limit DNA damage in the nervous system is obvious, delineation of disease pathogenesis is lacking. Understanding the basis for genome instability–related neurologic disease requires knowledge of the relevant pathogenic DNA lesions and subsequent cellular sequelae.

During neurogenesis, which is characterized by extensive and rapid DNA replication, maintenance of genome integrity requires DNA repair to resolve multiple types of replication-associated damage. For example, misincorporation of ribonucleotides, base mismatches, strand breaks, cross-links, and replication-transcription collision cause DNA damage during replication (*3*–*5*). DNA repair is also essential in the differentiated (mature) nervous system, where a qualitatively different spectrum of DNA lesions and signaling outcomes will occur. Further, in comparison to neurogenesis, DNA damage–induced apoptosis does not readily occur in mature/differentiated neural cells (*6*). In mature neural cells, the high oxidative load of the brain and generation of free radicals from cellular metabolism mean that oxidative DNA damage repair is paramount (*7*–*9*). Transcription-associated DNA damage is also a main source of genome instability, and this can occur from base damage, strand breaks, or aberrant topoisomerase activity that deregulate RNA polymerase activity (*6*, *10*, *11*). Transcription-associated R loops and other uncommon DNA structures such as G-quadruplexes are also increasingly being recognized as potential sources of genotoxic stress, particularly in the nervous system (*12*–*14*). R loops are an RNA-DNA hybrid formed between a nascent transcript and the template DNA strand, which generates a displaced DNA strand that is susceptible to damage (*15*). However, multiple genome maintenance factors suppress inappropriate R loop formation, and mutations in some, such as the senataxin helicase, can cause neurodegenerative disease (*10*, *12*, *13*, *16*). The ATM [ataxia telangiectasia (A-T), mutated] protein kinase has also been directly implicated in the prevention of R loops caused by ultraviolet (UV) transcription-blocking lesions that disrupt spliceosome organization (*17*). While R loops are recognized as a threat to genome integrity, much about the physiological events driving their formation in the context of pathology is unknown, particularly so in differentiated cells.

To understand pathogenesis associated with genome instability syndromes, we generated mouse models with chronic basal DNA damage via inactivation of neurodegenerative disease–relevant repair factors. We used *Atm^Nes-cre^* mice, a model for A-T, and engineered additional disruption of *Aptx* to increase genome instability in the nervous system. *APTX* mutations are causative in ataxia with oculomotor apraxia 1 (AOA1), a syndrome with similar cerebellar pathology to A-T. This attenuation of DNA repair in the double mutants lowered the threshold of genome instability, leading to a progressive marked ataxia in all double-mutant mice. These new models now provide a fundamental tool to explore how compromised genome instability affects the nervous system and how this causes progressive and profound neurodegeneration. Mechanistically, our data reveal that DNA damage selectively affects the Purkinje cells (PCs) of the cerebellum via perturbation of RNA polymerase kinetics and mRNA splicing, promoting R loops and genotoxicity, which drives cerebellar degeneration. Using genome-wide ATAC-seq (assay for transposase-accessible chromatin using sequencing), we find why these defects are restricted to cerebellar PCs by showing that an unusually open chromatin conformation, much more so than is usual for expressed genes, characterizes these susceptible gene loci. These findings reveal that cell type–specific chromatin structure may determine the pathogenesis of neurodegeneration.

## RESULTS

### Compromised DNA strand break repair leads to neuropathology and ataxia

**Fig. 1.**
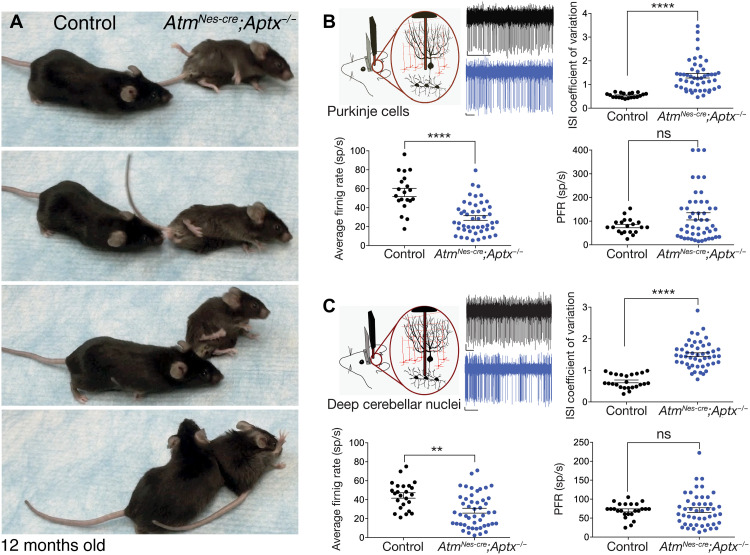
Inactivation of DNA repair factors result in progressive ataxia and cerebellar dysfunction. (**A**) Mice with dual inactivation of ATM and APTX (*Atm^Nes-cre^;Aptx^−/−^*) progressively develop a profound ataxia commencing from 6 to 7 months of age. Single-mutant (*Atm^Nes-cre^* or *Aptx^−/−^*) mice do not develop ataxia. Photo credit: Young Don Kwak, SJCRH. Schematic of in vivo extracellular recordings from PCs (**B**) and deep cerebellar neurons (**C**) in awake, head-restrained 12-month-old mice. Example raw traces are shown from control animals (black) and *Atm^Nes-cre^*;*Aptx^−/−^* mice (blue). Quantification of the average firing rate of PCs recorded from control mice and age-matched *Atm^Nes-cre^;Aptx^−/−^* mice shows a significant decrease in the double-mutant animals. The interspike interval (ISI) coefficient of variation (CV) of *Atm^Nes-cre^;Aptx^−/−^* animals was significantly increased compared with control cerebellum, indicating a highly irregular firing of PCs in double-mutant mice. The predominant firing rate (PFR) was not significantly different between control and mutants. sp/s, spikes/s; ns, not significant, **P < 0.01 and ****P < 0.0001.

While loss of the DNA damage–responsive protein kinase ATM (causal for A-T) or the DNA base excision repair factor APTX (aprataxin, a nucleotide hydrolase, mutated in AOA1) results in severe spinocerebellar ataxia, mouse models of these diseases fail to show overt neurodegeneration or behavioral phenotypes. A possible reason may be greater resistance of the rodent brain to DNA repair deficiency, particularly because *Atm^−/−^* mice fully recapitulate the extraneurologic features of A-T (*18*). To understand the pathogenic impact of neural genome instability in a disease-relevant context, we generated mice with coincident inactivation of the DNA repair factors APTX and ATM. *Atm^Nes-cre^*;*Aptx^−/−^* mice all developed progressive and substantial ataxia beginning at around 7 months of age (100% penetrance; *n* ≥ 30 animals) (Fig. 1A). We also generated *Atm^Nes-cre^*;*Parp1^−/−^* mice as poly(adenosine diphosphate–ribose) polymerase (PARP1), similar to APTX, which is also important for base excision repair (*19*), and these mice also developed an identical phenotype to *Atm^Nes-cre^*;*Aptx^−/−^* mice. Last, to distinguish the contribution of ATM inactivation to this phenotype, we also established *Aptx^−/−^*;*Parp1^−/−^* mutant animals, but these did not develop ataxia. An occasional *Aptx^−/−^*;*Parp1^−/−^* animal developed a late-onset gait phenotype characterized by dystonia and hind limb dysfunction, but they lacked overt ataxia. Notably, other combinations of compromised DNA repair such as DNA-PKcs (DNA-dependent protein kinase, catalytic subunit) or DNA ligase IV together with ATM loss do not develop ataxia (*20*, *21*). Our data establish that specific disease-relevant endogenous DNA lesions can compromise neural homeostasis, resulting in a loss of motor control and ataxia.

To understand the basis for ataxia present in the *Atm^Nes-cre^*;*Aptx^−/−^* and the *Atm^Nes-cre^*;*Parp1^−/−^* mice, we evaluated nervous system histology. Unexpectedly, general cerebellar histology via Nissl staining was unremarkable, even in strongly affected *Atm^Nes-cre^*;*Aptx^−/−^* or *Atm^Nes-cre^*;*Parp1^−/−^* animals, and showed no obvious cell loss to account for the neurologic deficits (fig. S1). Magnetic resonance imaging of the mutant and control brains showed a modest but significant reduction in the overall volume of the double-mutant cerebellum, despite overall normal histology (24% decrease; *P* < 0.0036; fig. S2). In addition, we observed a decrease in cortical volume (11% decrease; *P* < 0.001), and immunolocalization of cortical layers using FOXP2 (Forkhead box protein P2) and BRN2 (POU3F2); Brain-2 showed a modest reduction in neuronal numbers in the double-mutant cortex (fig. S3A). Myelination was also largely unperturbed in the double-mutant brain (fig. S3B). We also assessed DNA damage using γH2AX immunoreactivity to identify DNA double-strand breaks. While scant γH2AX was observed in control or single mutants, *Atm^Nes-cre^*;*Aptx^−/−^* showed obvious nuclear foci at 4 to 6 weeks of age, although γH2AX signal decreased substantially in older mice, possibly reflecting a different spectrum of DNA damage (e.g., DNA single-strand breaks, which would not generate γH2AX) (fig. S4). Thus, despite severe ataxia, the overall brain/cerebellar histology in the double-mutant mice was relatively normal.

### Cerebellar function is perturbed in the double-mutant mice

Because the double-mutant mice displayed a severe motor phenotype, we examined the spontaneous activity of individual PCs and deep cerebellar nuclei (DCN) neurons in the cerebellum of 12-month-old awake head-restrained mice. Using single-unit extracellular recordings, we found that the average firing rate of PCs in double-mutant mice was significantly decreased by almost 50% when compared to control mice (average firing rates: control, 64.3 ± 3.9 and 56 ± 4.3; *Atm^Nes-cre^*;*Aptx^−/−^*, 29.1 ± 2.5; *Atm^Nes-cre^*;*Parp1^−/−^*, 29.3 ± 3.4; means ± SEM; ****P < 0.0001) (Fig. 1B and fig. S5D). To determine the firing regularity of PCs, we measured the coefficient of variation (CV) of the interspike interval (ISI). Although the PCs in double-mutant cerebellum have a decreased average firing rate, the CV ISI of PCs recorded from both mutant animals was significantly increased compared to their littermate controls. This indicates that the activity of these cells is highly erratic (average CV ISI: control, 0.43 ± 0.02 and 0.54 ± 0.02; *Atm^Nes-cre^*;*Aptx^−/−^*, 1.37 ± 0.1; *Atm^Nes-cre^*;*Parp1^−/−^*, 1.1 ± 0.09; means ± SEM; ****P < 0.0001) (Fig. 1B and fig. S5, B to D). We also calculated the predominant firing rate (PFR; the reciprocal of the mode ISI), and although there was no significant difference between mutants and the controls, there were a few cells that had very high PFR, indicating high-frequency bursting in these cells (average PFR: control, 76.8 ± 4.4 and 79 ± 7; *Atm^Nes-cre^*;*Aptx^−/−^*, 120.2 ± 2; *Atm^Nes-cre^*;*Parp1^−/−^*, 77.8 ± 11; means ± SEM) (fig. S5, C and D).

The DCN constitute a major output from the cerebellum. In turn, PCs constitute the only source of cerebellar cortical input to the neurons of the DCN, being a critical determinant of DCN function. Because PCs were affected in the double-mutant animals, we recorded DCN neurons to determine whether their activity was also altered (Fig. 1C and fig. S5). Similar to PCs, the average firing rate of DCN neurons in the double-mutant mice was significantly decreased compared to the controls (average firing rate: control, 42.6 ± 4.7 and 44.5 ± 2.9; *Atm^Nes-cre^*;*Aptx^−/−^*, 28.4 ± 2.6; *Atm^Nes-cre^*;*Parp1^−/−^*, 18.2 ± 1.5; means ± SEM; **P < 0.01) (Fig. 1C and fig. S5). Cells in the DCN also fired irregularly, as shown by the increased CV ISI that was twice as high as the controls (average CV ISI: control, 0.53 ± 0.03 and 0.65 ± 0.04; *Atm^Nes-cre^*;*Aptx^−/−^*, 1.5 ± 0.06; *Atm^Nes-cre^*;*Parp1^−/−^*, 1.3 ± 0.08; means ± SEM; ****P < 0.0001) (Fig. 1C and fig. S5, B to D), while the PFR remained unchanged between the double-mutant and control animals (PFR: control, 57 ± 5.6 and 70.6 ± 4; *Atm^Nes-cre^*;*Aptx^−/−^*, 71 ± 6.2; *Atm^Nes-cre^*;*Parp1^−/−^*, 62.8 ± 11.9; means ± SEM; Fig. 1C and fig. S5, C and D). Thus, the in vivo electrophysiological findings in the *Atm^Nes-cre^*;*Aptx^−/−^* and *Atm^Nes-cre^*;*Parp1^−/−^* mice demonstrate that PCs and cells in the DCN fired irregularly and decreased average firing rates. These data suggest that the abnormal motor phenotype in the double-mutant mice involves aberrant output of the cerebellum.

### Disrupted cerebellar homeostasis results from gene expression perturbation

**Fig. 2.**
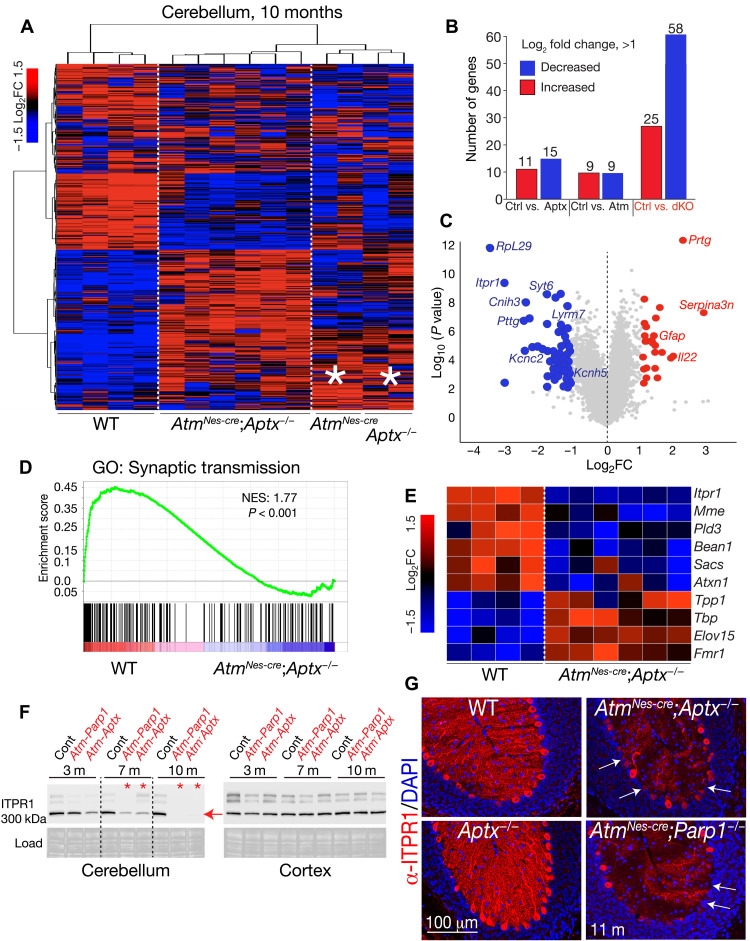
Disrupted cerebellar homeostasis in the DNA repair–deficient cerebellum results from perturbation of gene expression. (**A**) Heatmaps showing the top 1000 expressed genes from RNA-seq analysis from affected 10-month-old *Atm^Nes-cre^;Aptx^−/−^* cerebella (*n* = 6) show that a similar group of genes are decreased in expression. While there is a clear difference between gene expression in double-mutant cerebella compared to WT (*n* = 4), single *Aptx^−/−^* (*n* = 2), or *Atm^−/−^* (*n* = 2) mutants show a trend toward gene expression reflective of the double mutant, as indicated by asterisks. (**B**) A common set of 58 genes are decreased in double-mutant cerebella, while 25 genes are increased in expression. dKO, double knockout. (**C**) A volcano plot shows the change in gene expression compared to significance of expression change with representative gene names indicated. (**D**) An example of gene set enrichment analysis (GSEA) shows a significant decrease in genes required for synaptic function in the double-mutant cerebella. (**E**) Western blot analysis shows that ITPR1 levels decrease with age, and most ITPR1 is absent in the double-mutant cerebellum by 10 months of age. In contrast to the cerebellum, ITPR1 levels are not affected in the double mutant cortex. (**F**) Immunohistochemistry for ITPR1 shows a loss of PC expression in the 11-month-old double-mutant cerebellum compared to WT tissue. (**G**) In many PCs, expression of ITPR1 is markedly decreased/absent as indicated by arrows.

To further investigate the molecular basis for ataxia, we evaluated the transcriptome of *Atm^Nes-cre^;Aptx^−/−^* cerebellar and cortical tissue using RNA sequencing (RNA-seq). Using the total RNA (Ribo-Zero preparation) from symptomatic 10-month-old double-mutant mice and littermate controls, we determined the comparative levels of the top 1000 expressed genes in each genotype (Fig. 2A and table S1). The double-mutant cerebella samples showed a reproducible decrease in expression [log_2_ fold change (FC) > 1, false discovery rate (FDR) < 0.05] of a common group of ~58 genes compared with wild-type (WT) controls (Fig. 2B and table S1). Among these, the type 1 inositol 1,4,5-trisphosphate receptor (*Itpr1*) was markedly decreased in expression (Fig. 2C). Notably, inactivating mutations in *ITPR1* in both humans and mice result in spinocerebellar ataxia (*22*, *23*), illustrating the potential functional relevance of the affected genes to the cerebellar phenotype in the double-mutant mice. In both the single mutants (i.e., *Atm^−/−^* and *Aptx^−/−^*), gene expression changes in the cerebellum trend toward the expression patterns seen in the double-mutant mice, suggesting that a threshold effect is relevant for pathology (Fig. 2A, asterisks). We also compared gene expression differences (log_2_FC > 1) between the cortex of the *Atm^Nes-cre^;Aptx^−/−^* mice and controls and found 49 significantly altered genes (FDR < 0.05). Of these, only eight were also present in the cerebellum, reflecting the tissue-specific nature of the mutant phenotype. While cortical expression changes are possibly interesting, these have not been considered further in the current study.

Using gene set enrichment analysis (GSEA), we compared our double-mutant expression data with controls and observed that multiple pathways were affected in the mutant tissue. These include a lack of enrichment for synaptic activity, neuronal identity, apoptosis, and potassium channel function in the double-mutant cerebellum (Fig. 2D and fig. S6). We also compared the gene decreases in the double-mutant cerebellum with the DISEASE database (*24*), and text mining identified additional genes with reduced expression that are linked to ataxia (using a differential cutoff of FDR < 0.05; fig. S6). To functionally validate reduced gene expression in the double mutants, we used Western analysis to demonstrate the progressive loss of ITPR1 that, by 10 months of age, was substantially reduced in both the *Atm^Nes-cre^;Aptx^−/−^* and *Atm^Nes-cre^;Parp1^−/−^* cerebella. Conspicuously, ITPR1 loss was restricted to the cerebellum, as levels in the cortex were unchanged (Fig. 2E). Further, immunohistochemistry demonstrated a notable loss of ITPR1 in the 11-month-old *Atm^Nes-cre^;Aptx^−/−^* cerebellum, which showed an almost complete absence of immunostaining in the Purkinje dendrites and frequent loss in the cell body (Fig. 2F). Expression of other regulators of cerebellar homeostasis linked to human disease was also markedly decreased in double (but not single)–mutant cerebella including carbonic anhydrase VIII (CA8), whose loss results in ataxic waddles mice and cerebellar ataxia in humans (table S2 and fig. S7) (*25*, *26*). Notably, while the decrease in ITPR1 and CA8 is notable, this is not associated with PC loss, as indicated by direct quantification of these cells (fig. S7). Thus, a group of key homeostatic neuroregulators are consistently diminished in the cerebellum of DNA repair–deficient double-mutant mice.

### Aberrant RNA splicing of key cerebellar homeostatic genes in the ataxia mutants

Gene expression alterations in the double-mutant mice implicate chronic genotoxic stress affecting transcription, which drives pathogenesis. Direct links between DNA damage and transcription are known, particularly after UV damage (*11*), although the relevance and impact in a physiologic setting, especially in differentiated neurons, remain unknown. DNA damage can promote altered RNA splicing and decreased gene expression (*11*, *27*, *28*), and aberrant splicing is implicated in neurodegenerative diseases including amyotrophic lateral sclerosis, fragile X syndrome, and spinal muscular atrophy (*14*, *29*–*31*). Therefore, we assessed RNA splicing in a double-mutant brain tissue using multivariate analysis of transcriptional splicing (rMATS) (*32*) and a splicing deficiency score algorithm (*33*) to assess intron retention.

**Fig. 3.**
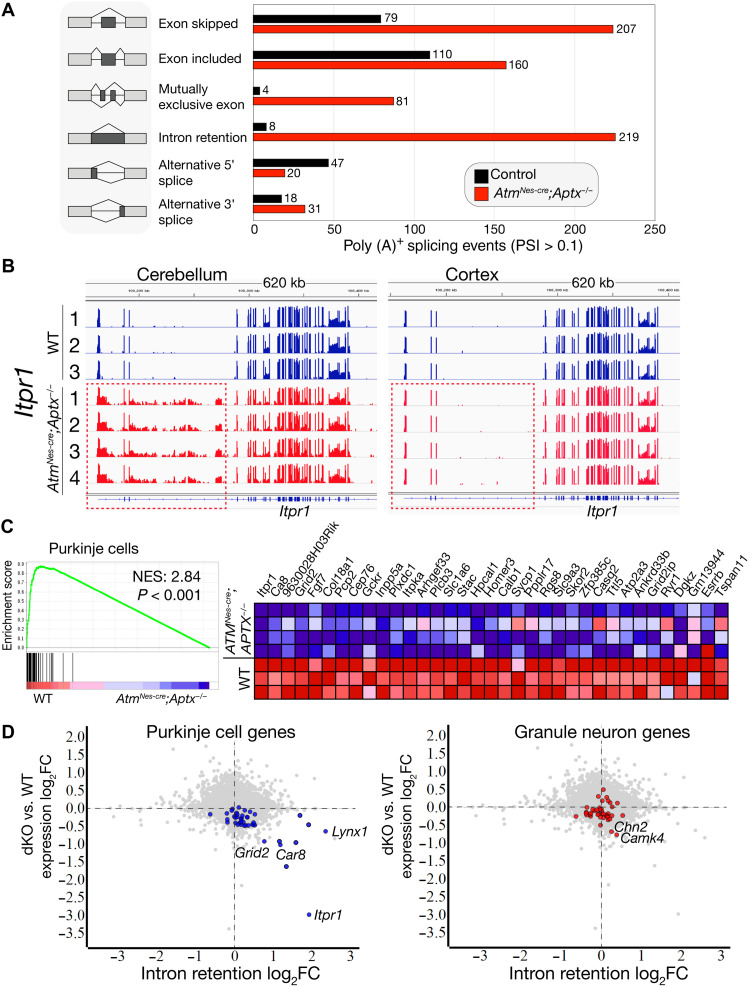
Aberrant RNA splicing in cerebellar PCs in the ataxia mutants. (**A**) Splicing defects occur in the *Atm^Nes-cre^;Aptx^−/−^* cerebellum. rMATS analysis (*32*, *77*) shows alterations in all classes of mRNA splicing in the double mutants. Additional identification of intron retention was performed using the splicing deficiency score algorithm (*33*). (**B**) An example of intron retention in *Itpr1* shows all mutant *Atm^Nes-cre^;Aptx^−/−^* have a similar recurring splicing abnormality in the cerebellum, but not in the cortex (red dashed boxes). (**C**) Gene set enrichment using a PC-specific gene set (*35*) shows that gene expression in these neurons is affected in the double-mutant cerebellum. The adjacent heatmap shows the expression of the PC genes in the double-mutant cerebellum compared to WT. NES, normalized enrichment scores. (**D**) Gene expression (mRNA-seq) compared to intron retention (splicing deficiency score) shows that PC genes feature high-intron retention and lower gene expression. Representative Purkinje and granule neuron–specific gene names are listed. The significant threshold of *P* < 0.05 was applied as a differential cutoff. The graphs indicate a strong correlation between intron retention and gene expression changes in PCs, but not granule neuron gene expression in the double mutants.

To increase the sensitivity of detecting variations in mRNA structure, we generated polyadenylated [poly (A)^+^] libraries for mRNA-seq from additional 12-month-old control and double-mutant brain tissue. By comparing mRNA-seq data from six individual double-mutant cerebella (four *Atm^Nes-cre^;Aptx^−/−^* and two *Atm^Nes-cre^;Parp1^−/−^*) with controls, we identified a combined 2222 unique genes with splicing alterations of 12,595 individual messages analyzed [FDR < 0.05, a percent-splice-in (PSI) > 0.1], indicating 17.6% of genes are potentially affected (table S2). We then did a batch comparison between double-mutant and control cerebella mRNA-seq data to ascertain whether the genes that have recurring splicing aberrations are also reduced in expression. After normalization to remove genes with splicing variants found in both WT and mutant samples, we found a consistent set of 647 genes in the *Aptx^−/−^;Atm*^*Nes-cr*e^ cerebella samples (631 genes in *Parp1^−/−^;Atm*^*Nes-cr*e^ cerebella) that were aberrantly spliced (Fig. 3A and table S3). Notably, many aberrantly spliced genes including those with decreased expression initially identified in double-mutant cerebellar tissue using Ribo-Zero RNA-seq (Fig. 2). In contrast to the double-mutant cerebellum, we did not observe widespread splicing defects in the single-mutant (*Atm^−/−^* or *Aptx^−/−^*) or *Aptx^−/−^;Parp1^−/−^* tissues.

Intron retention can be deleterious for gene expression; therefore, we examined genes whose expression was attenuated in the double-mutant cerebellum. Inspection of *Itpr1* showed evident retention of the 23-kb second intron in the *Atm^Nes-cre^;Aptx^−/−^* (and *Atm^Nes-cre^;Parp1^−/−^*) cerebellum (Fig. 3B). In contrast, analysis of cortical *Itpr1* revealed a normal mRNA structure, consistent with our finding of normal ITPR1 levels in the double-mutant cortex (see Fig. 2E). Notably, all double-mutant cerebellar tissue analyzed showed identical aberrant *Itpr1* splicing, indicating that a tissue- and locus-specific genomic event underpins this recurring abnormality. Other down-regulated genes in the double-mutant cerebellum were also characterized by intron retention, including *Afap1l1*, *Cntn4*, *Car8*, and *Lynx1*, which we visually inspected using an Integrated Genomics Viewer (IGV) and confirmed by quantitative polymerase chain reaction (qPCR) to identify retained introns using additional mutant cerebella samples at different ages (fig. S8). Similar to *Itpr1*, all aberrantly spliced transcripts generally exhibited an identical pattern of locus-specific intron retention (figs. S8 and S9). However, while splicing alterations were very similar across mutant samples, there are stochastic aspects to these events such as (apparently) normal processing of *Cntn4* in one mutant cerebellum despite that particular sample showing defective splicing for *Afap1l1*, *Ca8*, *Itpr1*, and *Lynx1* (fig. S8).

We also confirmed that other types of splicing defects were present in the double-mutant cerebellum (fig. S9). In many cases, alternative splicing events were observed from 3 months of age, indicating that these are chronic events in the double-mutant cerebellum. Gene ontology (GO) pathway analysis of the cerebellar genes affected by splicing abnormalities identified pathways linked to neurologic disease, synaptic transmission, and glutamatergic synapse function (table S2). Thus, in the double-mutant cerebellum, widespread abnormalities occur, involving a notable cohort of critical neuroregulatory genes including *Itpr1*, *Grid2*, and *Ca8*, whose perturbation in humans underpin ataxia syndromes (*22*, *25*, *26*, *34*).

### PC-specific RNA splicing defects characterize the ataxia mutants

Unexpectedly, although aberrant splicing events were frequent in the double-mutant cerebellum, our analysis does not support broad general splicing abnormalities in cerebellar transcripts (the Kolmogorov-Smirnov test of the distribution of splicing deficiency between WT and double-mutant cerebellum is *P* = 0.213). However, examination of the affected genes in the double-mutant cerebellum implicated PCs as a source of the most aberrantly spliced transcripts. Therefore, we examined mRNA-seq data for specific cerebellar cell types (*35*, *36*) and confirmed that transcripts with a diminished expression in the double-mutant cerebellum reflected a PC expression profile (Fig. 3C). Further, using a splicing deficiency score (*33*) to measure intron retention, we confirmed a significant association between increased intron retention and decreased gene expression in PCs of the double mutant (Fig. 3D). In contrast, we failed to find significant intron retention events in granule neurons, although a couple of granule cell–specific genes *Chn2* and *Camk4* did show intron retention (Fig. 3D). PC-specific gene expression of intron-retained transcripts including *Afap1l1*, *Ca8*, *Cntn4*, and *Grid2* was confirmed by in situ hybridization of an adult (P56) brain using the Allen Brain Atlas resource (http://mouse.brain-map.org/) (fig. S10) (*37*). While analysis of a PC-specific expression using the gene sets from Rosenberg *et al.* (*35*) showed that ~70% of these genes had intron retention, there are hundreds of additional genes with intron retention and reduced expression in the double mutant. Figure S11 illustrates that those 225 of 1076 genes with retained introns also have significantly decreased expression. Notably, because assignment of PC-specific gene expression is based on single-cell signatures of highly expressed genes [e.g., Purkinje late (*35*)], this underestimates the number of affected Purkinje-specific genes in the double mutants. Surveying additional genes in the double mutants with both intron retention and decreased expression identified many others with PC-specific expression, including *Aff1*, *Kcnip4*, *Slc1a3*, and *Stxbp2* (fig. S12 and table S2). Therefore, the double-mutant cerebellum is characterized by a broad perturbation of Purkinje neuron gene expression, which is frequently associated with retained introns.

Intron retention can generate premature termination codons that block gene expression and promote nonsense-mediated decay (NMD) (*38*, *39*). On the basis of the prevalence of intron retention in the double mutants, we determined whether NMD gene expression patterns occurred in the double-mutant tissues. GSEA analysis for NMD pathways, including 3′ mRNA end processing, growing transcript cleavage, and intron containing pre-RNA, was significantly enriched in the cerebellum, implicating this process in decreased gene expression (fig. S13). These data directly implicate transcriptional disruption associated with aberrant gene splicing of critical PC regulatory genes as a selective target of genotoxic stress.

### RNA polymerase II occupancy at promoters is reduced in double-mutant cerebellum

**Fig. 4.**
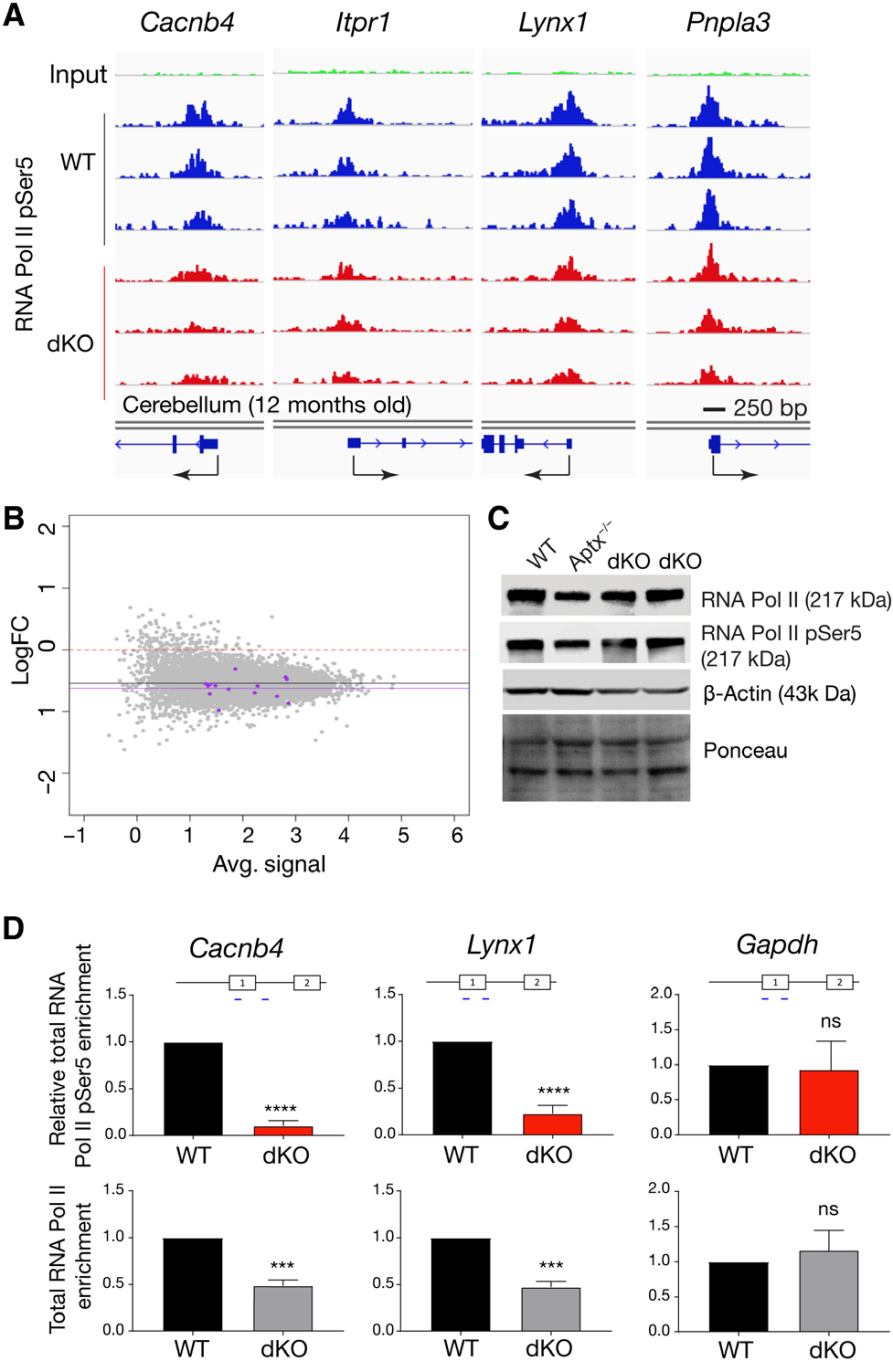
RNA Pol II promoter occupancy is reduced in the double-mutant cerebellum. (**A**) IGV visualization of comparative RNA Pol II binding after Pol II (pSer5) ChIP-seq of PC-specific loci from library size–normalized data using three independent 12-month-old WT and three *Atm^Nes-cre^;Aptx^−/−^* cerebella. Peaks found in Purkinje gene promoters show decreased RNA Pol II (pSer5) binding, and many including *Cacnb4*, *Lynx1*, and *Pnpla3* show a significant decrease when log_2_FC is adjusted *P* < 0.05. Gene promoters and exon/intron structure are indicated under the respective IGV image for each gene; arrows indicate the transcriptional start site and the direction of transcription. Each IGV track represents an independent tissue sample. (**B**) Plot of average signal of all samples versus log_2_FC between WT and knockout (KO) and from promoter/transcription start site (TSS) peaks identified in RNA Pol II (pSer5) ChIP-seq. The red dashed line at 0 log_2_FC indicates a mean representative of no change in promoter occupancy, but with overall RNA Pol II (pSer5) binding reduced in the double-mutant samples. The black line indicates the mean log_2_FC of all peaks at *x̅* = −0.54, while purple dots represent genes from the “Purkinje late” gene set (*35*), showing that their mean log_2_FC is lower than the general mean, *x̅* = −0.63, *P* = 0.13. (**C**) Western blots of cerebellar tissue extracts show similar levels of RNA Pol II and RNA Pol II (pSer5) in WT, *Aptx^−/−^*, and two times independent 12-month-old *Atm^Nes-cre^;Aptx^−/−^* tissue. (**D**) Quantitative analysis of RNA Pol II binding using ChIP-qPCR shows a decrease in bound RNA Pol II and a further decrease in RNA Pol II (pSer5) at promoter sites for PC-specific gene loci (*Cancnb4*, ****P < 0.001; and *Lynx1*, ***P < 0.01), but not at *Gapdh*.

Alterations in RNA polymerase II (Pol II) kinetics can affect key cotranscriptional processes such as normal splicing and transcript elongation and can influence NMD (*40*). Specific types of DNA damage can variably influence RNA Pol II activity. For instance, DNA damage arising from oxidative strand breaks and, to a lesser extent, base modifications can disrupt normal transcriptional processes (*41*, *42*). DNA double-strand breaks can also affect transcription in an ATM-dependent manner, while transcription-coupled repair effectively bypasses bulky DNA lesions/adducts (*11*, *43*). To determine whether chronic DNA damage affected RNA polymerase occupancy, we globally assessed RNA Pol II binding throughout the genome in the double-mutant cerebellum using chromatin immunoprecipitation sequencing (ChIP-seq) with antibodies recognizing RNA Pol II (pSer5). This RNA Pol II modification is enriched at promoters and linked to spliceosome activity (*44*). Using library size normalization, we found a reduction of between 20 and 45% in RNA Pol II (pSer5) occupancy at multiple Purkinje gene promoter regions in the double-mutant cerebellum (Fig. 4A). Global assessment of promoter occupancy using log_2_FC versus average signal showed a general reduction in occupancy at promoter regions in the double-mutant cerebellum (mean of −0.54), although Purkinje genes trend toward a further, but not significant, reduction in RNA Pol II occupancy (Fig. 4B). We also confirmed that protein levels of RNA Pol II and Pol II (pSer5) were similar between control and double-mutant cerebellum, indicating that differences in polymerase occupancy did not reflect broad alterations in enzyme levels across genotypes (Fig. 4C).

To quantify RNA Pol II occupancy, we used ChIP-qPCR of both RNA Pol II and RNA Pol II (pSer5) at representative PC gene loci (*Cacnb4* and *Lynx1*) and found a significant reduction in binding of both RNA Pol II and RNA Pol II (pSer5) (between *P* < 0.01 and *P* < 0.001), but not at *Gapdh* (Fig. 4D). Thus, normal kinetics of RNA Pol II occupancy is affected in the double mutant at the genome loci of PC genes showing transcriptional disruption. These data suggest that, in response to chronic genotoxic insult, RNA Pol II kinetics are perturbed, potentially contributing to the transcription and splicing abnormalities in the double-mutant cerebellum.

### R loops accumulate in the double-mutant cerebellum

R loops are a potentially genotoxic and pathogenic trihelical DNA structure in which the nascent transcript hybridizes to the template DNA strand, displacing the nontemplate strand (*10*, *13*, *15*, *45*, *46*). R loops play an important physiological role, (*16*), aberrant R loops can be associated with splicing abnormalities and, in this context, are posited to promote DNA damage (*15*, *16*, *47*).

**Fig. 5.**
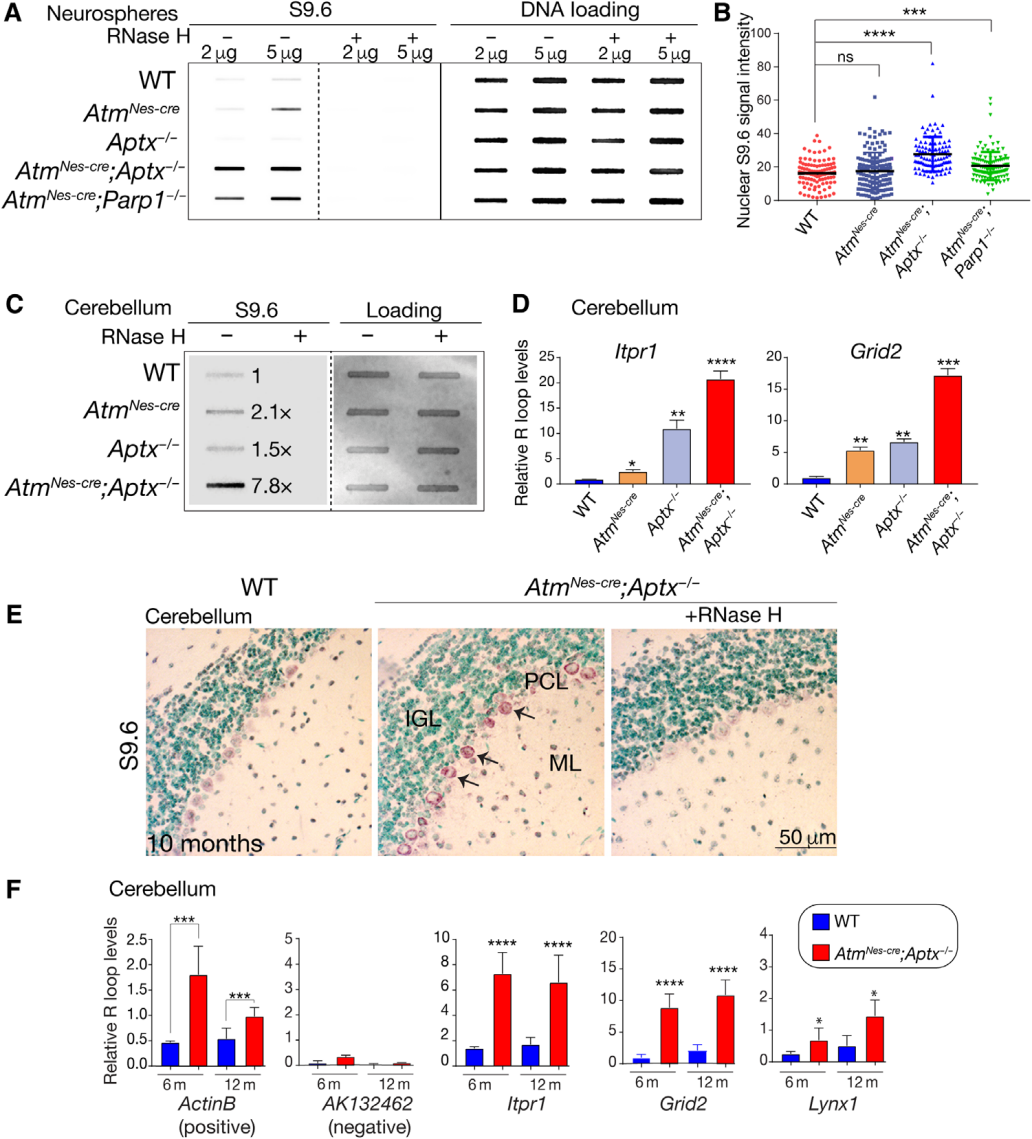
R loops occur in the double-mutant cerebellum. (**A**) Immunoblotting of isolated genomic DNA shows a high level of R loops in the double-mutant neurospheres. Specificity of the S9.6 antibody is confirmed as signal is absent after RNase H treatment of samples. DNA loading is shown using SYBL Gold nucleic acid stain. (**B**) Quantification of R loops in *Atm^Nes-cre^;Aptx^−/−^* and *Atm^Nes-cre^;Parp1^−/−^* neurospheres show high levels of nuclear R loops compared to controls identified via immunocytochemistry using the S9.6 antibody. (**C**) R loop formation in the cerebellum is substantially increased in the double-mutant tissue (~8×), which is abolished by RNase H treatment showing specificity of the S9.6 antibody. (**D**) DRIP-PCR analysis shows that R loops are present at elevated levels in *Itpr1* and *Grid2* in the double-mutant cerebellum. *P < 0.05, **P < 0.01, ***P < 0.001, and ****P < 0.0001. (**E**) Immunohistochemistry of cerebellar sections from control and mutant, using S9.6 to identify R loops. RNase H treatment confirms the specificity of the S9.6 antibody. IGL, inner granule layer; PCL, PC layer; ML, molecular layer. (**F**) DRIP-qPCR shows R loop levels in *Itpr1*, *Grid2*, and *Lynx1* in cerebellar tissues from the double-mutant compared to controls at 6 and 12 months of age. *ActinB* was used as a positive locus for R loops and *AK132462* as a negative control. *P < 0.05, ***P < 0.001, and ****P < 0.0001.

Therefore, to establish whether R loops co-occur with splicing abnormalities in the DNA repair mutants, we used the S9.6 antibody, which recognizes the characteristic DNA:RNA structure in R loops (*48*–*51*). We assessed global R loop formation via slot blotting of genomic DNA and found a clear increase in S9.6 binding in the double-mutant neurospheres compared to WT cells (Fig. 5A). We consistently found variable levels of elevated R loops in either of the *Aptx^−/−^* or *Atm^Nes-cre^* single-mutant samples. The elevated S9.6 signal was abolished with ribonuclease H (RNase H) treatment, confirming the DNA-RNA specificity of the S9.6 antibody. We also found a significant increase in S9.6 nuclear puncta in *Atm^Nes-cre^*;*Aptx^−/−^* and *Atm^Nes-cre^*;*Parp1^−/−^* neurospheres compared to WT and single-mutant progenitors (Fig. 5B). We then looked in vivo and examined double-mutant cerebellar tissue for R loop formation. We found an approximately eightfold increase in RNase H–sensitive S9.6 immunoreactivity in *Atm^Nes-cre^*;*Aptx^−/−^* cerebellar tissue compared to WT, showing that R loop formation occurs in vivo (Fig. 5C). Increased R loops were also observed in the single *Atm* and *Aptx* mutants, but at lower levels than the double-mutant tissue (approximately twofold versus approximately eightfold) (Fig. 5C). To determine whether R loop formation occurred in PC genes with intron retention in the double-mutant cerebellum, we examined specific gene loci using DNA-RNA immunoprecipitation (DRIP) with the S9.6 antibody. Using the *QmRLFS-finder* web server (*52*) to identify potential R loop locations in transcription start sites (TSSs) including the promoter and first intron regions, we found significant (*P* < 0.0001) elevation of R loops at *Itpr1* and *Grid2* loci in the 12-month-old double-mutant cerebellum, compared with single-mutant or WT tissue (Fig. 5D).

We also used immunohistochemical localization of S9.6 to assess R loop occurrence in cerebellar tissue sections from 12-month-old WT and *Atm^Nes-cre^*;*Aptx^−/−^* double mutants. Widespread immunolocalization of R loops to PCs was observed in the *Atm^Nes-cre^*;*Aptx^−/−^* double-mutant cerebellum, while little to no R loops were observed in the WT tissue (Fig. 5E). Pretreatment of *Atm^Nes-cre^*;*Aptx^−/−^* tissue with RNase H abolished positive S9.6 immunoreactivity, confirming signal specificity (Fig. 5E). R loop accumulation assessed over time revealed that, at 6 and 12 months in *Atm^Nes-cre^;Aptx^−/−^* cerebella, robust R loop formation occurred at *Itpr1*, *Grid*2, and *Lynx1* loci. *ActinB* was used as a positive control as it is known to accumulate R loops (*53*), while *AK132462* was used as a negative control (Fig. 5F) (*54*). R loops were also found in genes that showed exon skipping and are therefore not exclusive to only intron retention (fig. S14). These data indicate that R loop formation is characteristic of PC genes that have aberrant splicing and diminished expression in the double-mutant cerebellum.

### R loop formation is associated with splicing defects

Our data show that transcriptional disruption in the double-mutant cerebellum is characterized by both splicing defects and R loop formation of specific PC genes. To determine whether genotoxicity of R loops directly affect gene expression, we used SH-SY5Y cells, a human neuroblastoma line that expresses many cerebellar-relevant neural genes including *IPTR1*. We used two established RNA splicing inhibitors, pladienolide B (PladB) and isoginkgetin, which impairs U2 small nuclear ribonucleoprotein interaction with pre-mRNA and U4/5/6 recruitment to the spliceosome, respectively (*55*, *56*). While these inhibitors had little obvious effect on cell morphology or viability (fig. S15A), expression analysis showed a significant decrease in both *ITPR1*, *CACNB4*, *GRID2*, and *LYNX1* between 2 and 8 hours after treatment, while the intronless gene *H2AX* showed no change in expression (fig. S15B). PCR analysis of splicing inhibitor–treated SH-SY5Y cells showed exon alterations in *GRID2* and *CACNB4* transcripts (fig. S15C). We further confirmed global R loop formation after splicing inhibition by showing that S9.6 binding to genomic DNA was increased; this immunoreactivity was abolished by RNase H treatment, confirming antibody specificity (fig. S15D). Last, we found that PladB promoted R loops in *ITPR1*, *CACNB4*, and *snRNP* (a positive control for R loop formation) after 2 hours of treatment (fig. S15E). Thus, splicing inhibition can cause R loop formation and decreased gene expression at loci relevant to those in the double-mutant cerebellum.

### R loops induce DNA damage and directly interfere with gene expression

**Fig. 6.**
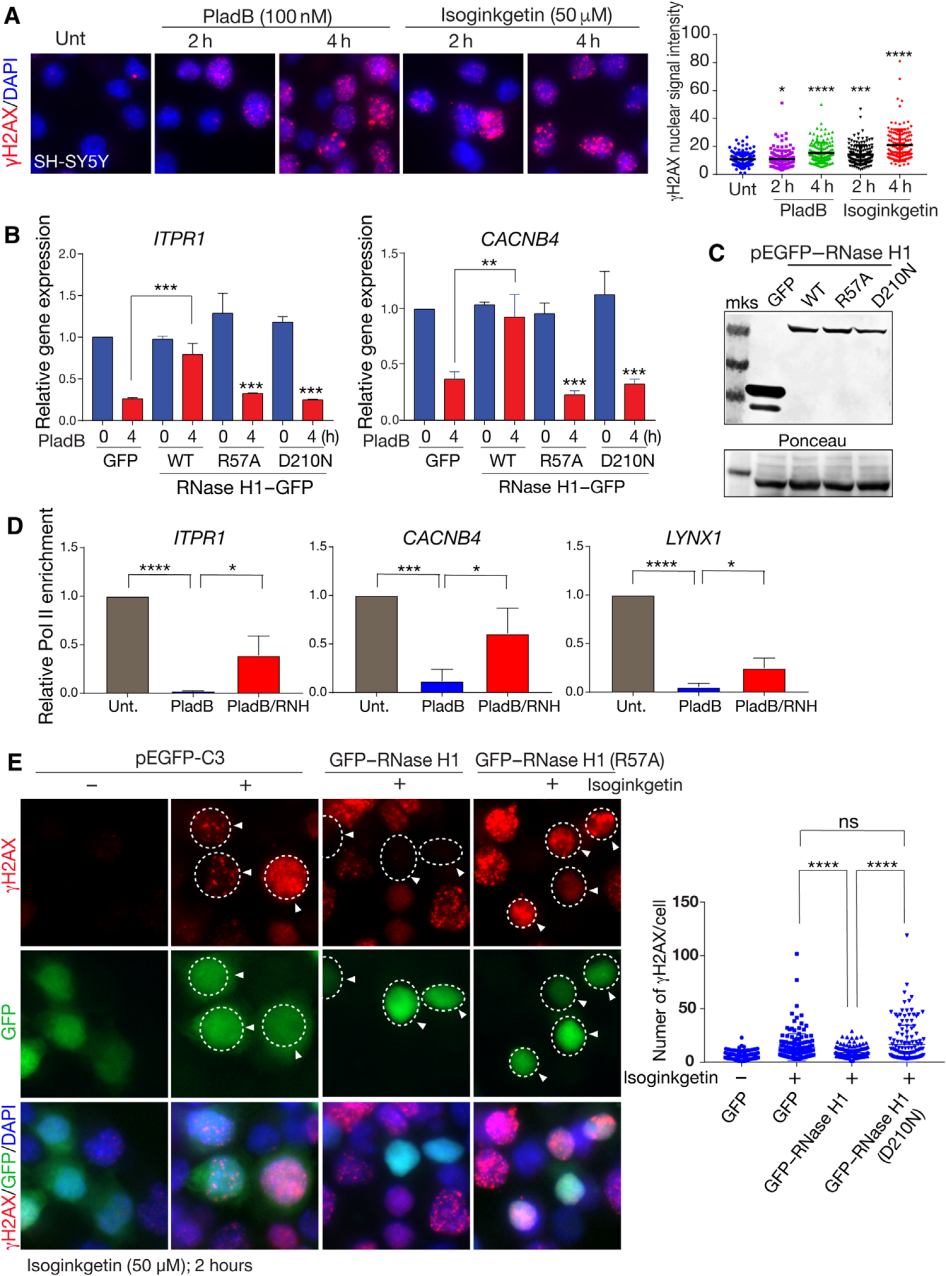
R loops result from defective splicing, causing DNA damage and inhibiting gene expression. (**A**) Splicing inhibition after isoginkgetin or PladB results in DNA damage as shown by γH2AX immunocytochemistry. Relative γH2AX levels are quantified in the adjacent plot. Unt, untreated control. (**B**) Inhibition of expression of *ITPR1* and *CACNB4* after PladB treatment in SH-SY5Y cells is relieved by RNase H1 but not a catalytic mutant (R57A) or RPA binding mutant (D210N). (**C**) Western blot analysis confirms equal expression of the WT, catalytic, and proliferating cell nuclear antigen–binding mutant versions of RNase H1. Ponceau staining shows equal protein loading. mks, Molecular Weight Marker. (**D**) Consistent with restoration of gene expression, RNA Pol II occupancy is also restored by RNase H1 (RNH) after PladB; relative Pol II enrichment after ChIP assessed by qPCR is shown for *ITPR1*, *CACNB4*, and *LYNX1*. (**E**) DNA damage after splicing inhibitor treatment (isoginkgetin) results from R loops, as γH2AX foci are abrogated by expression of WT RNase H1 but not the catalytic R57A mutant. *P < 0.05, **P < 0.01, ***P < 0.001, and ****P < 0.0001.

We found that splicing inhibitors caused significant levels of DNA damage (identified by γH2AX formation) at 2 hours after treatment, and, by 4 hours, more abundant DNA damage was observed (Fig. 6A). We considered whether these DNA strand breaks resulted from R loops associated with splicing inhibition, as there is a strong association between R loops and DNA damage (*10*). Therefore, we determined whether R loop formation was directly causal for gene expression decrease after splicing inhibition. Because RNase H1 can resolve R loops, we expressed RNase H1 or inactivating mutations of this enzyme, an R57A or D210N mutant RNase H1 where the catalytic activity or the required replication protein A (RPA) binding domain (*57*), respectively, was disrupted, to directly assess the impact of R loops. We found that decreased expression of *ITPR1* and *CACNB4* after splicing inhibition was recovered by WT RNase H1, but not with the R57A or D210N mutants, indicating that R loop formation after splicing inhibition affects gene expression (Fig. 6B). We also confirmed that the WT and mutant versions of RNase H1 were expressed at similar levels (Fig. 6C). Accordingly, RNase H1 treatment of cells after PladB also recovered RNA Pol II occupancy at promoters of genes affected by splicing disruption, including *ITPR1*, *CACNB4*, and *LYNX1* (Fig. 6D). These data indicate that R loop formation can suppress gene expression.

We next checked whether suppressing R loops by RNase H1 was sufficient to prevent DNA damage after splicing inhibition. We compared γH2AX formation after splicing inhibition in cells expressing WT RNase H1 or a D210N catalytic mutant. We found that expression of RNase H1 but not the D210N mutation was sufficient to abolish γH2AX formation after splicing inhibition, suggesting that R loops from aberrant splicing are genotoxic (Fig. 6E). To further confirm that R loops can affect cerebellar gene expression, we disrupted Aquarius (AQR), a DNA/RNA helicase that functions during splicing to suppress R loops (*57*–*59*). We used small interfering RNA inhibition of *AQR* and found that loss of this helicase causes a reduction in ITPR1 (fig. S16A). Accordingly, AQR inhibition also decreased *ITPR1* and *GRID2* gene expression, which was recovered by expression of AQR (WT), but not with an AQR helicase-dead (HD) Y1196A mutant (fig. S16, B and C). We then measured R loops at *ITPR1* and *GRID2* loci induced by PladB after WT or HD AQR expression and found that elevated R loops were selectively abrogated by expression of AQR, but not by the AQR HD mutant (fig. S16). Collectively, our findings illustrate that R loop formation occurs after splicing disruption, and this promotes DNA damage, elevates genotoxic stress, and contributes to decreased gene expression at specific genomic loci.

### Damage-susceptible gene loci in PCs are marked by a unique chromatin architecture

**Fig. 7.**
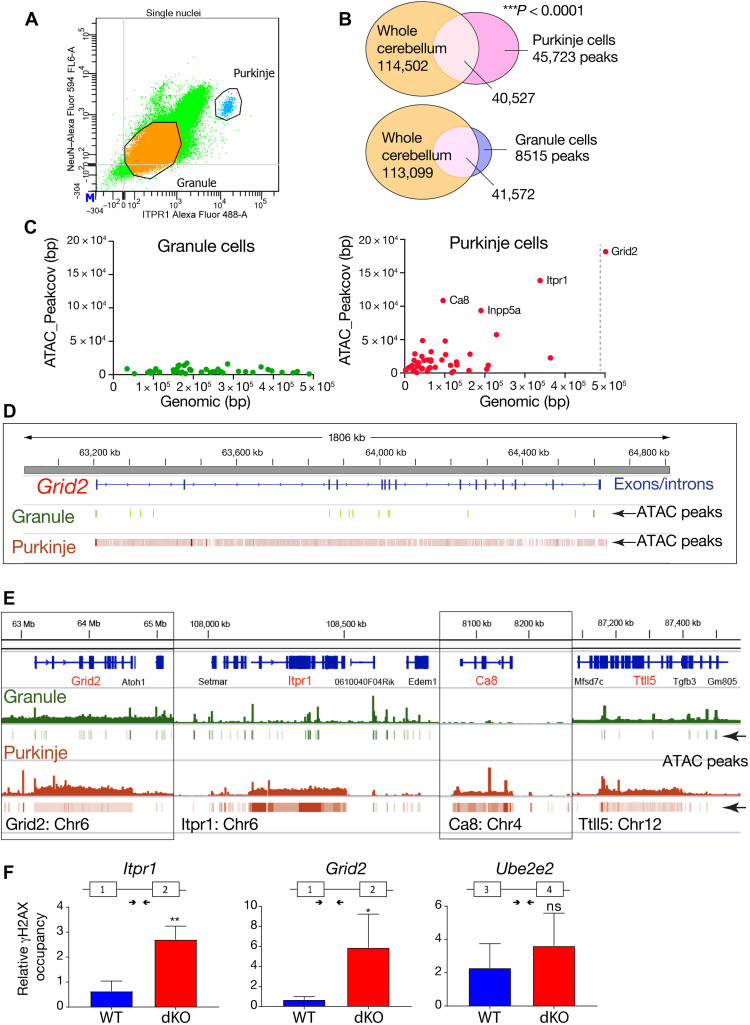
Global ATAC-seq profiling of Purkinje and granule neurons. (**A**) Purkinje and granule neuron nuclei from four pooled WT cerebella were isolated via flow cytometry, using NeuN and ITPR1, respectively. (**B**) The Venn diagrams show the overlap between MACS2 peaks in Purkinje or granule nuclei and whole cerebellum as determined by positional overlap using UpSetR. (**C**) Scatterplot showing the relationship between gene length and the ATAC coverage in GC and PC, respectively. Gene lists represent the most differentially expressed genes in PCs and GCs, as determined by Rosenberg *et al.* (*35*). Gene length in base pairs is shown on the *x* axis. The sum of the base pair length of all MACS2 peaks annotated to the gene by HOMER is shown on the *y* axis. (**D**) Representative IGV image of the distribution of ATAC peak at the *Grid2* locus in granule versus Purkinje neurons, showing the broad openness of the locus in PCs. (**E**) Whole-gene views of bigwig files and associated bed files of MACS2 peaks for granule and PCs. The comparison reveals the unique genomic open configuration across the entire gene body of *Grid2*, *Itpr1*, *Ca8*, and *Ttll5* in PCs. (**F**) γH2AX ChIP-qPCR shows elevated levels of γH2AX at *Itpr1* and *Grid2* loci in the double-mutant cerebellum (red) at 3 months of age. In contrast, *Ube2e2*, the expression of which is not altered in the double mutant, does not show significant differences in binding of γH2AX between WT (blue) and the double mutant (red). Experiments were done in triplicate; primer positions and exon structure are shown for each genomic locus. *P < 0.05, **P < 0.01, and ***P < 0.001.

PCs are large (>40 μm) and highly euchromatic, although detailed structural aspects of chromatin in these cells are lacking. PCs occur in low abundance within the cerebellum (~160,000 in mice), particularly compared to the large number of granule neurons (>10^7^ in mice), and thus need to be isolated for specific genomic DNA analysis. We used flow cytometry to isolate individual Purkinje and granule nuclei using ITPR1 and NeuN immunocapture from dissociated cerebella (*60*) and found that these represented 0.1 and 96.3% of the total population, respectively (Fig. 7A). We also confirmed PC-specific splicing abnormalities occurred in the double-mutant Purkinje neurons for *Abhd1*, but not in granule neurons, while *ActB* splicing was similar in both neuronal cell types and was unchanged in the mutant (fig. S17).

To determine whether the unique susceptibility of Purkinje neurons to DNA damage involves chromatin structure, we used ATAC-seq to globally profile the Purkinje genome. Initially, we compared ATAC-seq of purified Purkinje and granule neurons to that from the whole cerebellum. Overall, while ATAC peaks were found in multiple gene regions including the 3′ untranslated region (3′UTR), 5′UTR, exon, and transcriptional termination sites, ~98% of the peaks annotated to the promoter or TSS and shared overlap with peaks found in the whole cerebellum (table S4). Consistent with the abundance of granule cells, whole-cerebellum ATAC-seq data closely aligned with the isolated granule cell ATAC-seq (Fig. 7B). In contrast, PCs showed a substantially higher total number of unique ATAC peaks, with 45,723 unique peaks identified [spanning an average of 354 base pairs, as determined using Model-based Analysis of ChIP-seq2 (MACS2) peak-calling software], covering 28.15 million bases in protein-coding genes compared to granule cell nuclei, which showed much fewer overall unique peaks [8515 peaks with correspondingly longer peak length, averaging 856 base pairs (bp)], and spanning 16.3 million bases (Fig. 7B). This indicates that PCs have a chromatin accessibility profile, which is distinct from granule neurons.

To gauge the relevance and impact of the unique Purkinje ATAC-seq profile to the loss of gene expression and development of ataxia in the *Aptx^−/−^;Atm*^*Nes-cr*e^ cerebellum, we queried the chromatin structure at select gene loci affected in the double mutants. We examined WT cerebellum and initially used the Rosenberg gene sets (*35*) to assess the relative abundance and distribution of ATAC peaks in hallmark Purkinje and granule neuron genes, particularly as many of the genes on this list are affected in mutant PCs. Mapping peak coverage and genome length revealed that affected PC genes such as *Itpr1*, *Ca8*, and *Grid2* loci contain extraordinarily open chromatin, while similar regions of transposase-accessible chromatin were absent at these loci in granule neurons (Fig. 7C). More broadly, global ATAC-seq identifies a set of gene loci at different chromosomal locations in PCs with a highly open conformation, which contrasts granule cell (GC) chromatin organization (table S5). For example, 112 PC genes contain >20,000-bp peaks (of which 48 of these gene loci have >40,000-bp peaks), while only 10 genes in granule neurons contain >20,000-bp peaks (with none >40,000 bp) (table S5).

A detailed view of the peak coverage at specific Purkinje gene loci shows a dense array of peaks across the gene, spanning >10^6^ base pairs of DNA for *Grid2* in the PC genome (Fig. 7D). Lower-resolution examination of ATAC peaks indicate that these are densely distributed across the gene body, encompassing lengths ranging from 50 kb to 2 Mb, and occur throughout the Purkinje genome at loci affected in the double-mutant cerebellum (Fig. 7E and fig. S18). Analysis of select gene loci shows at least a 10-fold increase in the difference ratio (peaks/genome length) in PCs compared to GCs (fig. S19). However, the bulk of the PC genome shows relatively similar ATAC-seq profiles to that of granule neurons, emphasizing the unique chromatin structure at those loci affected by DNA damage (fig. S20).

To establish whether DNA damage associates with PC gene loci whose expression is affected in the double mutant, we used γH2AX ChIP. We found that DNA damage was elevated at affected PC gene loci (*Itpr1* and *Grid2*) in the double-mutant tissue (Fig. 7F). In contrast, *Ube2e2*, a gene unaffected in the mutants, showed no significant γH2AX accumulation (Fig. 7F). Thus, PCs have a highly open chromatin configuration, associated with many critical homeostatic genes, which renders them exceedingly susceptible to DNA damage.

## DISCUSSION

An ever-expanding group of human neurodegenerative diseases are characterized by DNA repair deficiency (*1*, *2*, *6*, *61*). These syndromes reflect the key roles played by multiple DNA damage response pathways to ensure neural homeostasis. However, the underlying basis for pathology in these syndromes remains largely unknown (*6*). Moreover, the age-related impact of DNA damage on the neural genome continues to be enigmatic, although aspects of brain aging have been ascribed to DNA damage accumulation (*2*, *62*, *63*). Thus, an outstanding question for a broad array of neurodegenerative syndromes, both congenital and age related, is that what are the pathogenic events that account for cell loss and/or neural dysfunction?

**Fig. 8.**
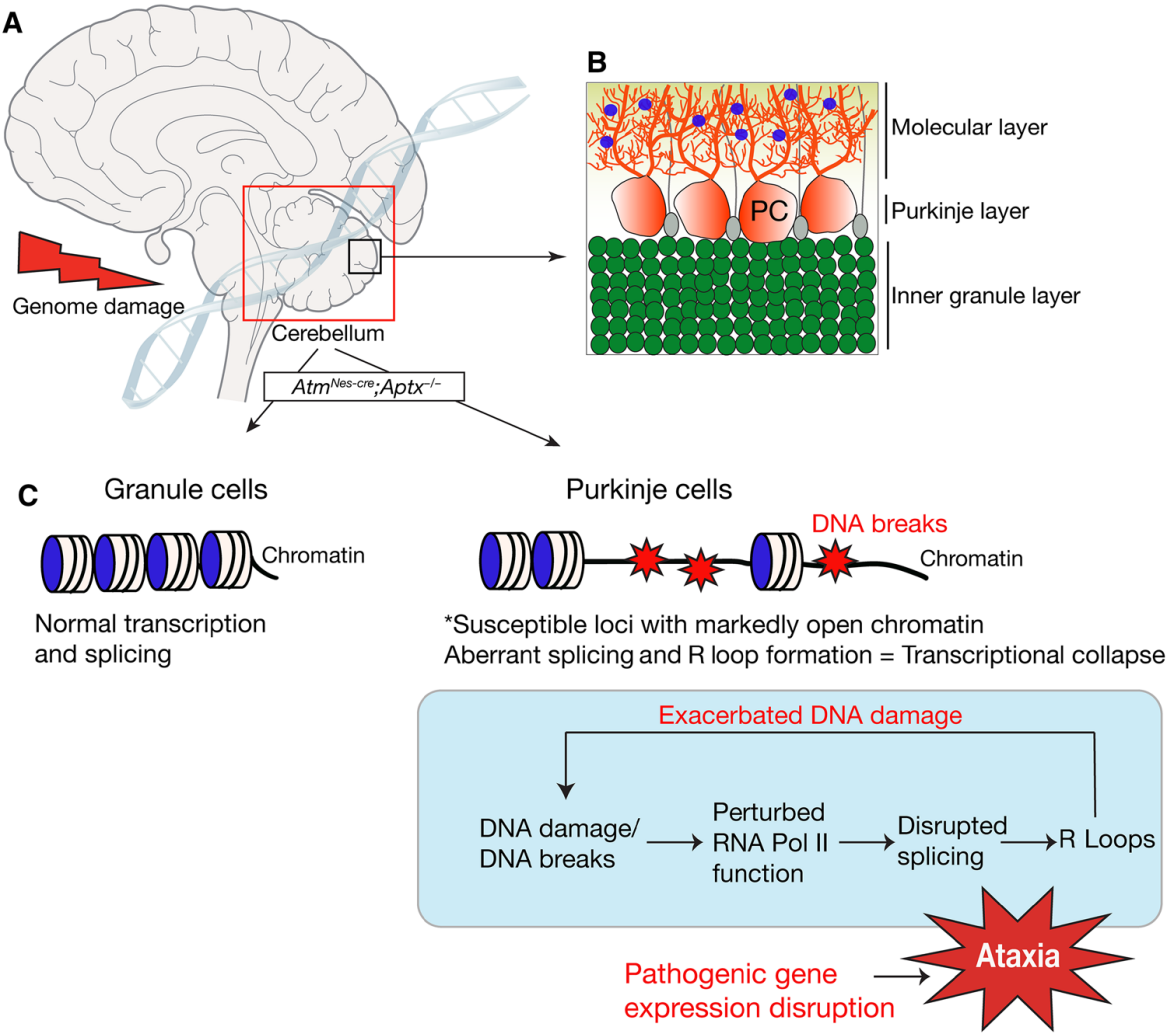
PC chromatin architecture dictates genotoxic stress susceptibility. The cerebellum is particularly affected by genotoxic stress. (**A**) Despite nervous system–wide damage in the case of inactivation of ATM and APTX, it is the cerebellum (boxed) that shows an overt defect. Within the cerebellum, the PCs are primarily affected by DNA damage. (**B**) The laminar structure of the cerebellum is shown (an expanded version of the smaller inner box of the cerebellar region) and indicates the three main layers: the inner granule layer, which contains the granule neurons; the Purkinje layer, which contains PCs (shown in red) interspersed with Bergmann glia cells (gray); and the molecular layer. The molecular layer contains PC dendrites that synapse with parallel fibers emanating from granule neurons and also interneurons (blue). (**C**) Granule cells and PCs have distinct chromatin architecture at certain gene loci. The architecture of these loci can predispose to DNA breaks that affect transcription by affecting RNA Pol II kinetics, causing splicing defects that lead to coincident R loop formation that can exacerbate DNA damage. Collectively, this detrimental effect on transcription leads to the pathogenic reduction of expression of critical homeostatic genes and subsequent PC dysfunction, resulting in ataxia.

In this study, we have shown that neurodegeneration in the double-mutant mice resulting from chronic DNA damage affects specific PC gene loci containing a unique chromatin architecture. Note that, while isolation of PCs is necessary to reveal chromatin architecture differences to GCs, bulk RNA-seq was able to discern gene expression differences in PC from the whole cerebellum. This likely reflects unique expression signatures of PCs, coupled with, in many cases, high gene expression levels. Consequently, genomic stress promotes transcriptional collapse of key neuroregulatory genes from abnormal splicing, particularly intron retention, and altered RNA Pol II occupancy at proximal promoter pause sites at these loci. As a result of aberrant splicing, genotoxic R loop formation is also greatly enhanced in these genes, which exacerbates DNA damage. These findings are summarized in Fig. 8. Thus, our data illustrate the consequences of defective genome stability that promotes pathogenic events that selectively (and severely) affect the transcriptional program in cerebellar PCs. These data have many important implications for understanding the bases for cellular dysfunction in neurodegenerative syndromes and the age-related effects of chronic genome damage.

Our study underscores the selective impact genotoxic stress has toward the PCs of the cerebellum. This vulnerable cell type is affected in A-T and in many spinocerebellar ataxias linked to genome instability including AOA1, AOA4, and SPINOCEREBELLAR ATAXIA, AUTOSOMAL RECESSIVE 26 (SCAR26) (*1*, *6*, *61*). In addition to our finding that a unique and highly open chromatin conformation mark susceptible Purkinje genes, there are other prominent aspects of PCs that compound this vulnerability to genotoxic stress. First, these are large cells compared to cerebellar granule neurons (~40 μm in diameter versus ~5 μm) and most other neurons in the brain. In this regard, it is known that large cells require a greater transcriptional activity to support basic cellular functions that proportionally increase the probability of genotoxic events arising from normal cellular activities (*64*, *65*). Thus, the high transcriptional activity of large neurons such as PCs and their requirement to function for the life of the organism may exacerbate the effects of chronic genotoxic stress. Second, PC chromatin is highly euchromatic (*66*), and analysis of DNA damage from ionizing radiation and radiomimetic drugs shows γH2AX accumulation preferentially in euchromatin (*67*, *68*). In this regard, it will be of interest to investigate chromatin architecture in other neural cells, particularly those with large nuclei and high transcriptional activity and pathogenic potential, such as motor neurons or oligodendrocytes for similar genotoxic stress susceptibilities as PCs.

DNA damage perturbs RNA Pol II kinetics, which has been linked to altered message-processing and -splicing abnormalities (*27*, *40*, *47*). Our finding of reduced Pol II promoter occupancy across the genome indicates that genotoxicity can globally affect polymerase kinetics, potentially causing pausing/trapping along the gene body (*27*, *40*, *42*), revealing that disrupted transcription is a consequence of DNA damage. We find that splicing aberrations disrupt the expression of many key cerebellar PC genes, many of which (e.g., *Itpr1*, *Grid2*, *Ca8*, *Bean1*, etc.) can individually compromise cerebellar function and lead to ataxia (*22*, *26*, *34*, *69*). In other scenarios of cerebellar dysfunction after DNA damage, ITPR1 has been reported to be decreased in a murine model of *Atm* and DNA *Pol*β inactivation (*70*) and has been also implicated in protein aggregation in A-T cerebellar pathology (*71*). Nonetheless, despite the direct connections of these individual genes to disease, our data do not necessarily point to any one gene as being responsible for the ataxic phenotype. Most likely, the PC/cerebellar dysfunction results from multiple transcriptional defects that collectively contribute to ataxia. R loops can arise in conjunction with abnormalities in mRNA splicing, and genotoxicity can result from breaks in the displaced DNA loop (*10*, *15*, *47*, *72*). We find elevated R loops highly enriched in PCs in the double-mutant cerebellum, and inhibition of RNA splicing or the inactivation of key R loop regulators such as RNase H1 and AQR markedly increases R loop–associated DNA damage. Notably, the aberrant RNA processing is highly recurrent, with identical intron processing defects across multiple individual double-mutant cerebella, which may reflect the effect of DNA damage toward specific chromatin architecture.

Collectively, our data show that chromatin architecture is linked to the susceptibility of gene loci to DNA damage, thus explaining the unique impact of this stress toward PCs. This, coupled with high transcriptional activity and robust oxygen metabolism of the nervous system, makes these cells a particularly susceptible target in genome instability syndromes.

## METHODS

### Animals

Conditional *Atm* (c*Atm*) and *Aptx* mutant mice and genotyping have been described (*73*, *74*); *Parp1^−/−^* and *Nestin-cre* mice were purchased from the Jackson Laboratory (stock nos. 002779 and 003771). We used c*Atm* (*Nestin-cre*) mice to restrict ATM deletion to the nervous system (to avoid thymic tumors that can occur in *Atm^−/−^* mice and synthetic lethality between dual loss of ATM and other repair factors (*75*). Mice of either sex were used for experiments, although, when possible, littermates were sex-matched. All datasets were derived from at least three individual animals. Mice were maintained in an American Association for Accreditation of Laboratory Animal Care (AAALAC)-accredited facility, and the St. Jude Institutional Animal Care and Use Committee approved all experimental work.

### Histology

Mice were perfused transcardially with 4% paraformaldehyde (PFA), while embryos were drop-fixed in 4% PFA. Tissue was cryoprotected in 25% phosphate-buffered saline (PBS)–buffered sucrose solution, embedded in optimal cutting temperature compound (OCT) and sectioned sagittally at 10 μm using an HM-500 M cryostat (Microm). Immunohistochemistry was performed after antigen retrieval (HistoVT One, Nacalai, USA). The following antibodies were used: anti-ITPR1 (1:500; Invitrogen, PA1-901) and anti–phospho-H2AX-Ser^139^ (1:200; Cell Signaling Technologies, #2577); the S9.6 antibody (1:500) was generated from the S9.6 hybridoma (American Type Culture Collection, HB-8730) or purchased from Kerafast (ENH001). Immunostaining of the S9.6 antibody was visualized with a VIP substrate kit (Vector Laboratories) and biotinylated secondary antibody and avidin-biotin complex (VECTASTAIN Elite kit, Vector Laboratories). Sections were counterstained with 0.1% methyl green (Vector Laboratories), dehydrated, and mounted with DPX (Fluka). For fluorescence, fluorescein isothiocyanate– or Cy3-conjugated secondary antibody (Jackson Immunologicals) was used and counterstained with 4′,6-diamidino-2-phenylindole (DAPI) or propidium iodide (Vector Laboratories). At least three independent tissue samples from each genotype were used for all experiments and analyses.

### Cerebellar electrophysiology

Mice were anesthetized with isoflurane and implanted with a small aluminum bracket fixed onto the skull with bone screws and Metabond (Parkell). A recording chamber was positioned on top of the cerebellum at coordinates anterior-posterior (AP): −6.2 mm and medial-lateral (ML): ±1.75 mm from the bregma. The craniotomy was surrounded with dental cement (M&S Dental Supply) and covered with a silicone elastomer, Kwik-Sil (World Precision Instruments). After 24 hours of recovery, mice were head-fixed using their implanted bracket to the headpost mounted on the stereotaxic holder. Mice were acclimated on the recording setup for 2 hours before the recordings were started. Single-unit neural activity was recorded extracellularly using a tungsten electrode (Thomas Recording, 2 to 3 megohms), which was slowly advanced into the cerebellum to target PCs and neurons of the DCN. Cells were identified on the basis of stereotaxic coordinates and the presence of complex spikes, which is a characteristic of PCs. Signals were band pass–filtered (200 Hz to 20 kHz), amplified (2000×), and digitized (20 kHz) using a National Instruments BNC-2110 and acquired using a custom-written software in LabVIEW 6.1. Waveforms were sorted offline (Plexon). Spiking characteristics such as amplitude, energy, and principal components analysis were used to sort cells into single units, where necessary.

### RNA sequencing

Total RNA was isolated from the tissue with TRIzol (Invitrogen) and library preparation using the TruSeq Stranded Total RNA Kit (Illumina). Double-stranded cDNA fragments were ligated with Illumina paired-end adaptors, followed by size selection (~200 bp), and libraries were analyzed by HiSeq 2000 sequencing systems (Illumina). FASTQ sequences were mapped to the University of California Santa Cruz (UCSC) mouse reference genome (mm9), and the gene-level fragments per kilobase of transcript per million (FPKM) values were computed for genome-wide analysis of differentially expressed genes via LIMMA in R 3.0.1. An unsupervised hierarchical clustering was used to generate the top 1000 most variable genes, which are selected using a median absolute deviation score. Statistical analyses and visualizations were performed using Partek Genomics Suite 6.6 and Stats MP/11.2 software. RNA-seq reads were mapped by an in-house mapping pipeline STRONGARM developed for the Pediatric Cancer Genome Project (*76*). Reads were aligned to four different databases using the Burrows-Wheeler Aligner (BWA) (0.5.10): (i) the mouse MGSCv37 reference sequence, (ii) RefSeq, (iii) a sequence file representing all possible combinations of nonsequential pairs in RefSeq exons, and (iv) the AceView database flat file downloaded from the UCSC Genome Browser, representing transcripts constructed from mouse ESTs.

### RNA splicing analysis

For the splicing analysis, reads were remapped using the STAR 2.5.0b (citation), and rMATS software (v.3.0.8) (*77*) was used to call the alternative splicing events. Specifically, we used “-t paired -cstat 0.0001” along with mouse GTF NCBIM37 file version 67. Splicing events reaching the significance level of FDR < 0.05 were reported. For capturing intron retention events, we calculated a splicing deficiency score that calculates a length-normalized intron-to-exon ratio. Genes with splicing defects were captured through LIMMA (*78*) and were manually validated using IGV. Splicing deficiency score = (no. of intronic reads/total intronic reads)/(no. of exonic reads/total exonic reads). We used a mock comparison between control versus control samples, which consists of a leave-one-out comparison for each of the four CNTRL samples (one versus the other three). Therefore, each APTX/ATM sample was compared against four control samples using a filter of *P* < 0.05 and PSI > 0.1 to identify aberrantly spliced events.

### Differential gene expression

Read counts for each gene was obtained with HTSeq (*79*). After the reads were normalized to FPKM, differential expression analysis was performed using LIMMA software (*78*). GSEAs were carried out using MSigDB (*80*). Specifically, we used “-metric signal2noise -set_min 4 -permute gene_set.” The GSEA FDR of 25% cutoff was applied to examine enriched gene sets.

### Allen Brain Atlas resource

Gene expression determination via in situ hybridization analysis was obtained from mouse P56 sagittal brain sections at the Allen Brain Atlas: https://mouse.brain-map.org/.

### Cell culture

SH-SY5Y neuroblastoma cells were grown in Dulbecco’s modified Eagle’s medium (DMEM) supplemented with 2 mM l-glutamine, penicillin (20 U/ml), streptomycin (20 mg/ml), and 15% (v/v) fetal bovine serum (Gibco, Gaithersburg, MD, USA). Cells were maintained at 37°C in a saturated humidity atmosphere containing 95% air and 5% CO_2_. Cells were treated with spliceosome inhibitors PladB (100 nM) (Sigma-Aldrich) or isoginkgetin (50 μM; Sigma-Aldrich) for 2, 4, and 8 hours.

Embryonic day 13.5 brains were isolated in cold PBS and 2% glucose for generation of neurospheres. After removing meninges, cerebral hemispheres were dissociated by mechanical trituration (20 strokes with a 1000-μl pipette and then 20 strokes with a 200-μl pipette) in NeuroCult NSC basal media (STEMCELL Technologies), supplemented with NeuroCult NSC proliferation media (complete media). To make a single-cell suspension, cells were further dissociated with a sterile fire-polished glass pipette (20 strokes). Cells were then resuspended in complete media supplemented with recombinant human epidermal growth factor (EGF) (20 ng/ml) and transferred to T25 flask, placed upright, and maintained at 37°C in a saturated humidity atmosphere containing 95% air and 5% CO_2_.

### Plasmids

#### Construction of pEGFP–hRNase H1, pEGFP–hRNase H1^D210N^, and pEGFP–hRNase H1^R57A^

The DNA containing the open reading frame of human RNase H1 [M27 RNase H1 isoform (*81*)] was PCR-amplified from a cDNA library prepared from SH-SY5Y cells using forward primer 5′-ACTCAGATCTCGAGCTCAAGATGTTCTATGCCGTGAGG-3′ and reverse primer 5′-GTCGACTGCAGAATTCGATCAGTCTTCCGATTGTTTAG-3′. The amplified DNA was cloned into the pEGFP-C3 vector using the Gibson cloning method [New England Biolabs (NEB)]. pEGFP–hRNase H1^D210N^ and pEGFP–hRNase H1^R57A^ plasmids (*57*) were constructed by introduction of D210N or R57A mutations in *EGFP-hRNase H1* using a QuikChange Lightning Multi Site-Directed Mutagenesis kit (Agilent), and mutations were confirmed by sequencing. Cells were plated in six-well plates at 80% confluence per well and, on the following day, were transfected with 4 μg of plasmids using Lipofectamine 2000 (Gibco-BRL, Carlsbad, USA). Cells were maintained in DMEM containing 10% fetal bovine serum and cultured for 48 hours.

### Western blots

Western blot analysis was performed with tissue extracts (cortex and cerebella) from mice of various genotypes. Protein extracts were prepared by using lysis buffer [50 mM tris-HCl, 200 mM NaCl, 0.2% NP-40, 1% Tween 20 (v/v), 1 mM NaF, 1 mM sodium vanadate, 50 mM β-glycerophosphate, 2 mM phenylmethylsulfonyl fluoride, and protease and phosphatase inhibitor cocktails (Roche)] and quantified by Bradford assay (Bio-Rad). Proteins (20 to 50 μg per lane) were separated through a 4 to 12% (w/v) bis-tris SDS polyacrylamide gel (Invitrogen) and transferred onto the nitrocellulose membrane (Bio-Rad). Blots were immunostained with the following antibodies: anti-ITPR1 (1:1000; rabbit; Invitrogen, catalog no. PA1-901) and anti–green fluorescent protein (GFP) (1:3000; rabbit; Life Technologies, catalog no. A11122), followed by appropriate horseradish peroxidase (HRP)–conjugated secondary antibodies (1:5000; GE Healthcare), and detected using a WesternSure PREMIUM chemiluminescence substrate (LI-COR) and a LI-COR Odyssey Fc (LI-COR). Anti-actin (1:2000; goat; Santa Cruz Biotechnology, clone I-19, catalog no. sc-1616) and Ponceau staining of the transferred membrane were used as protein-loading controls. Experiments were performed in triplicate.

### Polymerase chain reaction

Total RNA was extracted from brain tissues (cortex and cerebellum) and neuroblastoma SH-SY5Y cells using TRIzol (Thermo Fisher Scientific). RNA (1 μg) was reverse-transcribed to generate first-strand cDNA using the SuperScript III (Invitrogen, #18080). For reverse transcription qPCR, 2 μl of 1:5 dilution of the cDNA was used per well in a 20-μl reaction, along with 0.4 μl of 10 μM primer sets and 10 μl of the PowerSYBR Green PCR Master Mix (Thermo Fisher Scientific). Reactions were run in triplicate on an Applied Biosystems 7500 Real-Time PCR Machine (Applied Biosystems) with the following protocol: 50°C (2 min), 95°C (2 min), 40 cycles of 95°C (15 s), and 60°C (1 min). Sequences of all primers used in this study are detailed in the Supplementary Materials.

### S9.6 slot blots

Total nucleic acid was extracted by a standard SDS/proteinase K lysis, followed by phenol/chloroform extraction and ethanol/sodium acetate precipitation. DNA from each sample was spotted on a Hybond-N^+^ membrane (GE Healthcare) using a slot blot apparatus (Bio-Rad). For RNase H treatment, DNA was treated with RNase H (NEB) at 37°C overnight and then purified by standard phenol/chloroform ethanol precipitation. Membranes equilibrated with 25 mM NaPO_4_ (pH 6.5) were UV cross-linked (0.12 J/m^2^), blocked in 5% milk/Tris-buffered saline with 0.1% Tween® 20 detergent (TBST), and incubated overnight at 4°C with mouse S9.6 (1:1000). Blots were washed three times with phosphate-buffered saline solution with Triton X-100, and a secondary antibody (1:5000; goat anti-mouse HRP) was added for 1 hour at room temperature. The chemiluminescent signals were measured using LI-COR Odyssey Fc (LI-COR). As a loading control, the membrane was stained with a Diamond nucleic acid dye (SYBR) (Promega) or SYBR Gold nucleic acid gel stain (Invitrogen), and DNA was detected by LI-COR Odyssey Fc (LI-COR).

### Chromatin immunoprecipitation sequencing

Cerebellar tissues were collected from three WT and three *Atm^Nes-cre^*;*Aptx^−/−^* mice (10 to 12 months old) for RNA Pol II (pSer5) ChIP-seq. Frozen tissues were powdered, suspended in PBS containing 1% PFA to cross-link, pelleted, and then washed with ice-cold PBS containing protease inhibitor cocktail (Sigma-Aldrich, P8340). Chromatin was extracted in lysis buffer [0.25% Triton X-100, 1 mM EDTA (pH 8.0), 140 mM NaCl, 50 mM Hepes (pH 7.9), 10% glycerol, and 0.5% NP-40] and then washed [in 1 mM EDTA (pH 8.0), 200 mM NaCl, 10 mM tris-HCl (pH 8.0), and 0.5 mM EGTA (pH 8.0)]. Chromatin was then sheared [in 0.1% SDS, 1 mM EDTA (pH 8.0), and 10 mM tris-HCl (pH 8.0)] to generate DNA fragments of 200 to 500 bp using a Covaris M220 sonicator (Covaris). RNA Pol II C-terminal repeat domain (CTD) repeat YSPTSPS (phospho-S5) antibody (5 μg; Abcam, #ab5131) and the Magna ChIP Protein A + G Magnetic Beads (20 μl; Millipore, #16-663) were added to the chromatin and incubated overnight at 4°C. Beads were washed 1× with low-salt ChIP wash buffer [0.1% SDS, 1% Triton X-100, 2 mM EDTA, 20 mM tris-HCl (pH 8.0), and 150 mM NaCl], 1× with high-salt wash buffer [0.1% SDS, 1% Triton X-100, 2 mM EDTA, 20 mM tris-HCl (pH 8.0), and 500 mM NaCl] and 1× with LiCl wash buffer [1 mM EDTA, 10 mM tris-HCl (pH 8.0), 250 mM LiCl, 1% NP-40, and 1% sodium deoxycholate], and lastly in TE buffer [1% SDS, 1 mM EDTA, and 10 mM tris-HCl (pH 8.0)]. Immunoprecipitated DNA was eluted using 0.1 M NaHCO_3_/1% SDS, followed by addition of 4 μl of 5 M NaCl_2_ per IP to dissociate DNA-protein complexes, and incubated at 65°C overnight. For library preparation, immunoprecipitated DNA was quantified using the Quant-iT RiboGreen assay (Life Technologies). Libraries were prepared with HyperPrep Library Preparation Kits (Roche, PN07962363001) and analyzed for insert size distribution on a 2100 Bioanalyzer High Sensitivity kit (Agilent Technologies), 4200 TapeStation D1000 ScreenTape assay, or the Caliper LabChip GX DNA High Sensitivity Reagent Kit (PerkinElmer). Libraries were quantified using the Quant-iT PicoGreen dsDNA assay (Life Technologies) or low-pass sequencing with a MiSeq nano kit (Illumina). Single-read 50-cycle sequencing was performed on a NovaSeq 6000 (Illumina). For RNA Pol II ChIP-PCR, total Pol II was identified using an anti-RNA Pol II (Abcam, #ab26721) and for RNA Pol II (pSer5) (Abcam, #ab5131).

### ChIP-seq peak alignment and IGV visualization

The ChIP-seq raw reads were aligned to the mouse reference genome (mm9) using BWA (version 0.7.12; default parameter) and then marked duplicated reads with Picard (version 1.65). Only nonduplicated reads kept by SAMtools (version 1.3.1; parameter “-q 1 -F 1024”) were used for downstream analysis. R package SPP (version 1.11) was used to calculate relative strand correlation and to estimate the fragment size. We used genomeCoverageBed (bedtools 2.25.0) to produce genome-wide coverage in bedGraph file and then converted it to bigwig file by bedGraphToBigWig. The bigwig file was scaled by ChIP-seq library size to allow comparison across samples and was visualized by IGV (version 2.3.82).

### ChIP-seq peak calling

MACS2 (version 2.1.1) was used to call RNA Pol II (pSer5) peaks by comparing the IP library file to input library file with default parameters and an FDR cutoff of 0.05. Peak regions were defined to be the union of peak intervals in both WT and knockout (KO). Promoters were defined as mouse RefSeq TSS ± 1000-bp regions. Genomic feature annotation of peaks was performed by annotatePeaks.pl, a program from HOMER suite (v4.8.3; http://homer.ucsd.edu/homer/).

### ChIP-seq differential analysis

Raw read counts were reported for all peaks by bedtools (version 2.25.0) and then were voom-normalized and statistically contrasted using the LIMMA package (*78*) in R (version 3.3.1). An empirical Bayes fit was applied to contrast KO samples to WT samples and to generate log FCs, *P* values, and FDRs for each peak region.

### ChIP-qPCR analysis

To investigate DNA damage at PC gene loci, we performed γH2AX ChIP-qPCR. Chromatin was isolated from two pooled cerebella each of the 3-month-old WT and *Atm^Nes-cre^;Aptx^−/−^* mice, and γH2AX-bound DNA was precipitated using mouse anti–phospho-histone H2A.X (Ser^139^) antibody, clone JBW301 (Millipore, 05-636-I), as described above. The enrichment of γH2AX at *Itpr1*, *Grid2*, and *Ube2e2* was determined using qPCR analysis. To investigate the role of RNase H upon transcription regulation, pEGFP–hRNase H1 was transfected in SH-SY5Y cells. After 72 hours, 500 nM PladB was treated for 3 hours in untransfected and pEGFP–hRNase H1–transfected cells. Once chromatins were isolated, total RNA Pol ll ChIP was performed using anti-RNA Pol II CTD repeat YSPTSPS antibody (ChIP Grade, ab26721). Relative total RNA Pol ll enrichment at *Itpr1*, *Grid2*, and *Cacnb4* was determined and normalized with percent input, and fold difference was calculated. Supplementary Table 6; primer information. Experiments were performed in triplicate.

### DRIP and qPCR analysis

Nucleic acids were isolated from neurospheres (~2 × 10^6^ cells) and mouse cerebellum (~25 mg). Total nucleic acids were extracted with 0.5% SDS/proteinase K treatment at 37°C overnight and recovered by phenol-chloroform extraction and ethanol precipitation. Then, DNA was digested by a restriction enzyme cocktail (20 U of each Eco RI, Hind III, Bsr GI, Xba I, and Ssp I) (NEB). Fragmented DNA was cleaned by phenol-chloroform extraction and ethanol precipitation, followed by two washes with 70% ethanol and resuspended at 8 μg/ml in TE; 50 μl was saved as input for qPCR. Fifty microliters of 10× IP buffer was added (final buffer concentration of 10 mM sodium phosphate, 140 mM sodium chloride, and 0.05% Triton X-100) and incubated with 10 μl of S9.6 antibody for 16 hours at 4°C. Antibody-DNA complexes were bound to protein A/G agarose beads (Thermo Fisher Scientific) for 3 hours and washed thoroughly three times with 1× IP buffer. The bound DNA-RNA hybrid fractions were eluted [10 mM EDTA, 0.5% SDS, and 50 mM tris (pH 8)] and treated with proteinase K for 45 min at 55°C. Digested DRIP samples were phenol-chloroform–extracted, ethanol-precipitated, and resuspended in 50 μl of 10 mM tris-HCl (pH 8). As a control for S9.6 specificity, nucleic acids were treated with RNase H enzyme (NEB) overnight at 37°C. qPCR was performed at regions predicted to form R loops. The relative abundance of R loops at each loci was normalized to the signal of sample input.

### Granule and PC nuclei isolation

Nuclei were isolated according to Xu *et al.* (*60*). Isolated pooled cerebella tissue from four WT mice were collected and snap-frozen using liquid nitrogen and then thawed on ice for 30 min. Cerebella were then transferred to 5 ml of homogenization buffer [0.25 M sucrose, 150 mM KCl, 5 mM MgCl_2_, 20 mM tricine-KOH (pH 7.8), 0.15 mM spermine, 0.5 mM spermidine, 5 U of EDTA-free protease inhibitor cocktail, 1 mM dithiothreitol (DTT), Superase-In RNase inhibitor (20 U/ml), and RNasin ribonuclease inhibitor (40 U/ml)] and homogenized by 30 strokes with a glass dounce homogenizer. Subsequently, 4.6 ml of a 50% iodixanol solution [60% iodixanol/OptiPrep, 900 mM KCl, 30 mM MgCl_2_, 120 mM tricine-KOH (pH 7.8), 0.15 mM spermine, 0.5 mM spermidine, 4 U of EDTA-free protease inhibitor cocktail, 1 mM DTT, Superase-In RNase inhibitor (20 U/ml), and RNasin ribonuclease inhibitor (40 U/ml)] was added to tissue homogenates. Tissue homogenates were then added to a cushion of 2 ml of 27% iodixanol solution in the bottom of 13.2 ultraclear ultracentrifugation tube (Beckman Coulter, #41121703). Ultracentrifugation was performed at 10,000 rpm for 45 min at 4°C to isolate nuclei in a swinging bucket rotor (SW 41) in a Beckman Coulter XL-70 ultracentrifuge.

Nuclei pellets were carefully resuspended with 500 μl of homogenization buffer and fixed with 1% formaldehyde for 8 min at room temperature. Fixation was stopped by adding 0.125 M glycine for 5 min. Then, fixed nuclei were centrifuged at 1000*g* for 4 min, and the supernatant was discarded. Pellets were washed twice with wash buffer [PBS, 0.05% Triton X-100, bovine serum albumin (BSA; 50 ng/ml), 1 mM DTT, and Superase-In RNase inhibitor (10 U/μl)]. Nuclei were blocked with a blocking buffer [PBS, 0.05% Triton X-100, BSA (50 ng/ml), 1 mM DTT, Superase-In RNase inhibitor (10 U/μl), and BSA (50 ng/ml)] for 30 min. Then, nuclei were incubated with NeuN antibodies (1:250; mouse; Millipore, MAB377) and ITPR1 antibodies (1:500; rabbit; Invitrogen, PA1-901) for 16 hours at 4°C. The next day, nuclei were washed three times with a wash buffer and secondary antibodies (1:500) for 30 min at room temperature. Alexa Fluor 594 (mouse; ab150116) and Alexa Fluor 488 (rabbit; ab150077) were used as secondary antibodies. Right before flow cytometry sorting, nuclei were stained with 20 μM final concentration of DyeCycle Ruby (Invitrogen, V10273) for 10 min at room temperature. Cell type–specific nuclei were independently collected using a BD Biosciences FACSAria cell sorter, with excitation at 488, 561, and 640 nm. Gating on single nuclei using width versus area signals for DyeCycle Ruby was followed by gating of cell type–specific nuclei on the basis of NeuN and ITPR1 signals.

### ATAC-seq library generation and sequencing

Flow-sorted granule and PC nuclei (5000 each) described above were incubated for 10 min in nuclei lysis buffer [10 mM tris-HCl (pH 7.5), 10 mM NaCl, 3 mM MgCl_2_, and 0.05% NP-40], centrifuged for 5 min at 500*g*, and resuspended in 50 μl of transposition reaction mix [25 μl of 2× TD buffer, 2.5 μl of Nextera TDE1 enzyme (Illumina, #20034197), and 22.5 μl of nuclease-free water]. The transposition reaction was incubated at 37°C for 30 min with mild shaking. Then, 200 μl of reverse cross-linking solution [50 mM tris-HCl, 1 mM EDTA, 1% SDS, 0.2 M NaCl, and proteinase K (5 ng/ml)] was added to transposition reactants for overnight incubation at 65°C in a ThermoMixer (Thermo Fisher Scientific) with shaking at 800 rpm. DNA was then purified with QIAquick MinElute columns (QIAGEN, #28206) and amplified by PCR containing the Q5 High-Fidelity Polymerase (NEB, M0491S) for 12 cycles with ATAC-seq–barcoded primers. PCR reaction conditions were 72°C for 5 min; 98°C for 30 s; 12 cycles of 98°C for 10 s, 63°C for 30 s, and 72°C for 1 min; and 65°C for 5 min, with a hold at 4°C. Amplified libraries were purified and size-selected with AMPure XP beads (Beckman Coulter) and then sequenced on an Illumina HiSeq 4000, yielding 100-bp paired-end reads.

### ATAC-seq alignment, peak calling, and annotation

Trim Galore (version 0.4.4) was used to trim 3′ adapters from ATAC-seq raw reads with a quality score cutoff of Q20. Then, reads were aligned to the mouse reference genome (mm9) using BWA (version 0.7.12; default parameter). Duplicate reads were marked and removed by Picard (version 1.65). Only properly paired uniquely mapped reads were extracted by SAMtools (version 1.3.1). Mitochondrial reads were excluded from all downstream analysis. All mapped reads were offset by +4 bp for the + strand and −5 bp for the − strand. Last, nucleosome-free fragments (<100 bp) were extracted for calling peaks and generating bigwig files. Narrow peaks were called by MACS2 (version 2.2.7.1) with the following parameters: -q 0.01 -nomodel -extsize 200 -shift 100 -keep-dup all. Peaks were annotated using annotatePeaks.pl from HOMER software (version 4.8.3). The bigwig file was generated by UCSC tools, and IGV (2.3.82) was used for visual exploration of data.

## Notes

View/request a protocol for this paper from *Bio-protocol*.
