# Supplementary material for: Chromatin architecture at susceptible gene loci in cerebellar Purkinje cells characterizes DNA damage–induced neurodegeneration
Source: Sci Adv. 2021 Dec 15;7(51):eabg6363. doi: 10.1126/sciadv.abg6363 (PMC11323782; doi:10.1126/sciadv.abg6363)
Supplement: Supplementary file 1 — Figs. S1 to S20 Legends for tables S1 to S6 [file sciadv.abg6363_sm.pdf]

Science Advances
AAAS

Supplementary Materials for

Chromatin architecture at susceptible gene loci in cerebellar Purkinje cells characterizes DNA damage–induced neurodegeneration

Young Don Kwak, Timothy I. Shaw, Susanna M. Downing, Ambika Tewari, Aditi, Hongjian Jin, Yang Li, Lavinia C. Dumitrache, Sachin Katyal, Kamran Khodakhah, Helen R. Russell, Peter J. McKinnon*

*Corresponding author. Email: peter.mckinnon@stjude.org

Published 15 December 2021, *Sci. Adv.* **7**, eabg6363 (2021)
DOI: 10.1126/sciadv.abg6363

The PDF file includes:

Figs. S1 to S20
Legends for tables S1 to S6

Other Supplementary Material for this manuscript includes the following:

Tables S1 to S6

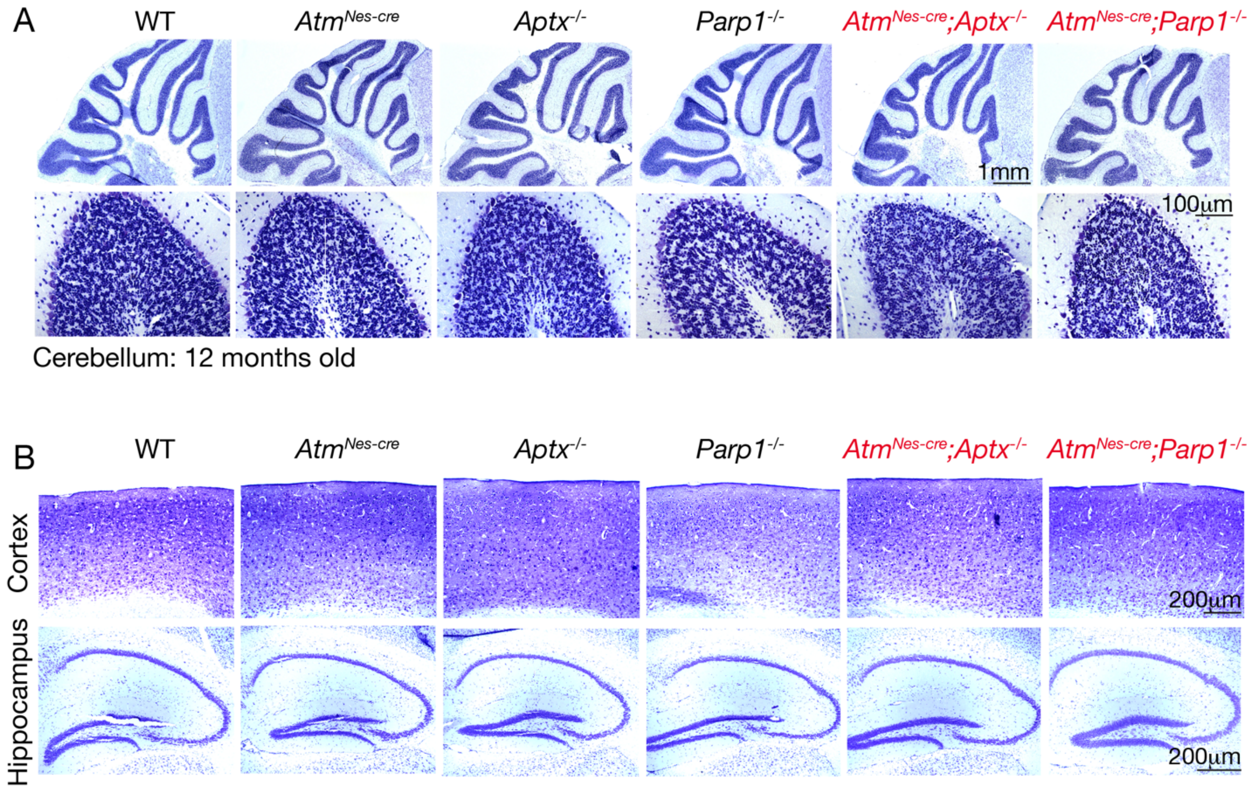

Supplemental Figure 1: Normal overall brain histology in mutant ataxic mice. (A) Nissl staining of 12-month-old affected (ataxic) double mutant (*Atm*$^{Nes\text{-}cre}$*;Aptx*$^{-/-}$ and *Atm*$^{Nes\text{-}cre}$*;Parp1*$^{-/-}$) mice show little overt or discernable histology to account for the profound ataxia. Upper panels show lower power sagittal images of the affected cerebellum of double mutants in comparison to WT and single mutants. Lower panels show the internal granule cell layer of the comparative genotypes. (B). Nissl-stained images of the cortex and hippocampal region from the same mice in (A) are shown.

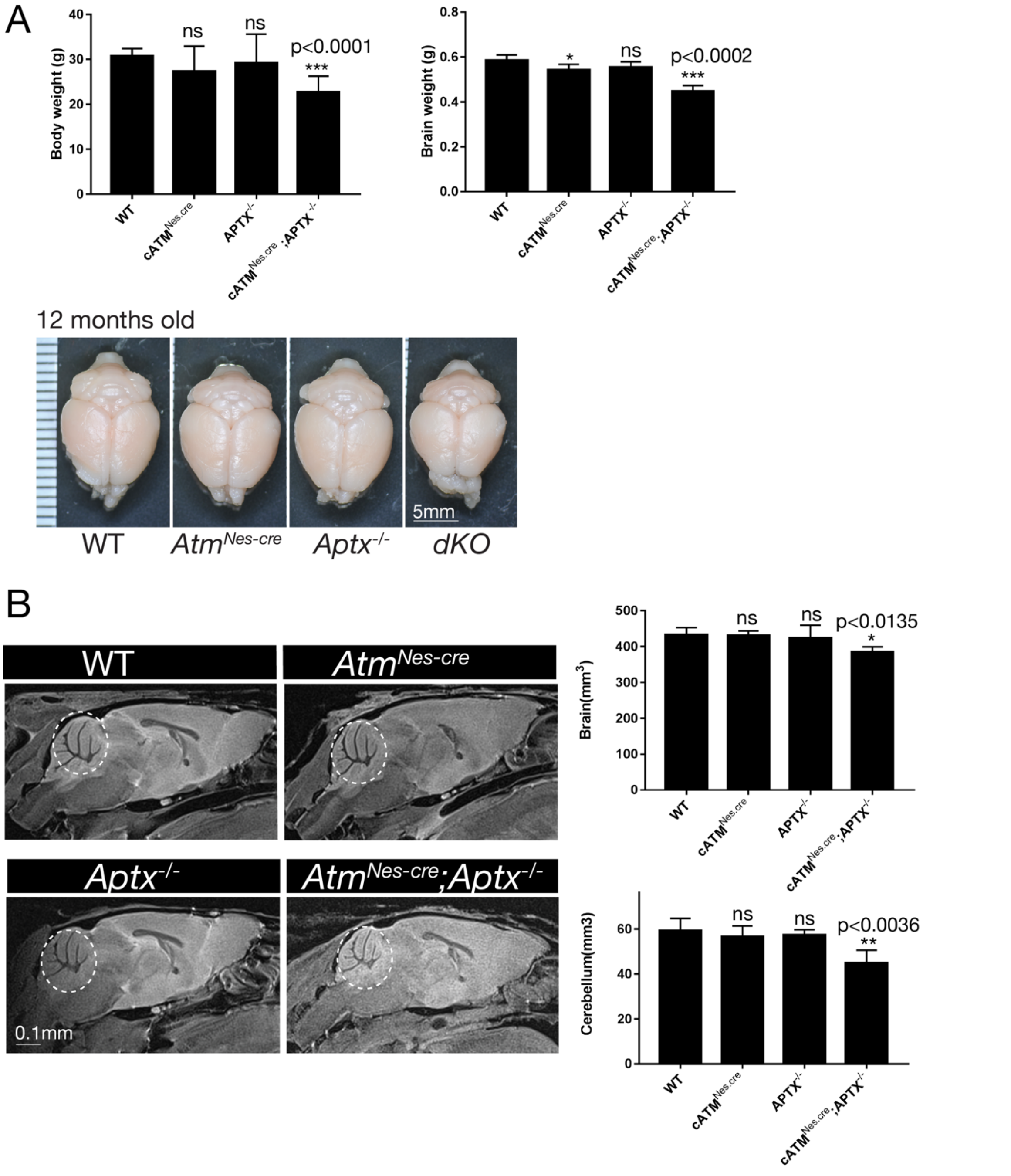

Supplemental Figure 2: Mild size decreases in the double mutant brain. (A) Analysis of brain weight from 12-month-old WT, single- or double mutant animals reveals a significant decrease in overall brain weight ($p<0.002$) and body weight ($p<0.0001$) in the double mutant. Photo credit: Yang Li, SJCRH. (B). MRI analysis shows a similar trend with a significant ($p<0.0036$) decrease in cerebellar volume (mm^3; white dashed circles), and mildly in overall brain volume ($p<0.0135$). MRI was performed using a 7 T Bruker

ClinScan system (Bruker BioSpin MRI GmbH, Germany) equipped with 12S gradient coil. A 2-channel surface coil was used for MR imaging. Animals were anesthetized and maintained with 1.5-2% isoflurane during MRI sessions. Transverse T2-weighted turbo spin echo images were acquired for volume measurements (TR/TE = 3660/50 ms, FOV = 25 x25, matrix = 320x320, NEX = 1, Thickness = 0.4 mm, scan time = 6.5 min). For data analysis, volume measurements (brain, olfactory bulb and cerebellum) were obtained by manually segmenting the regions and computing volumes using OsiriX (v5.7, Pixmeo, Switzerland).

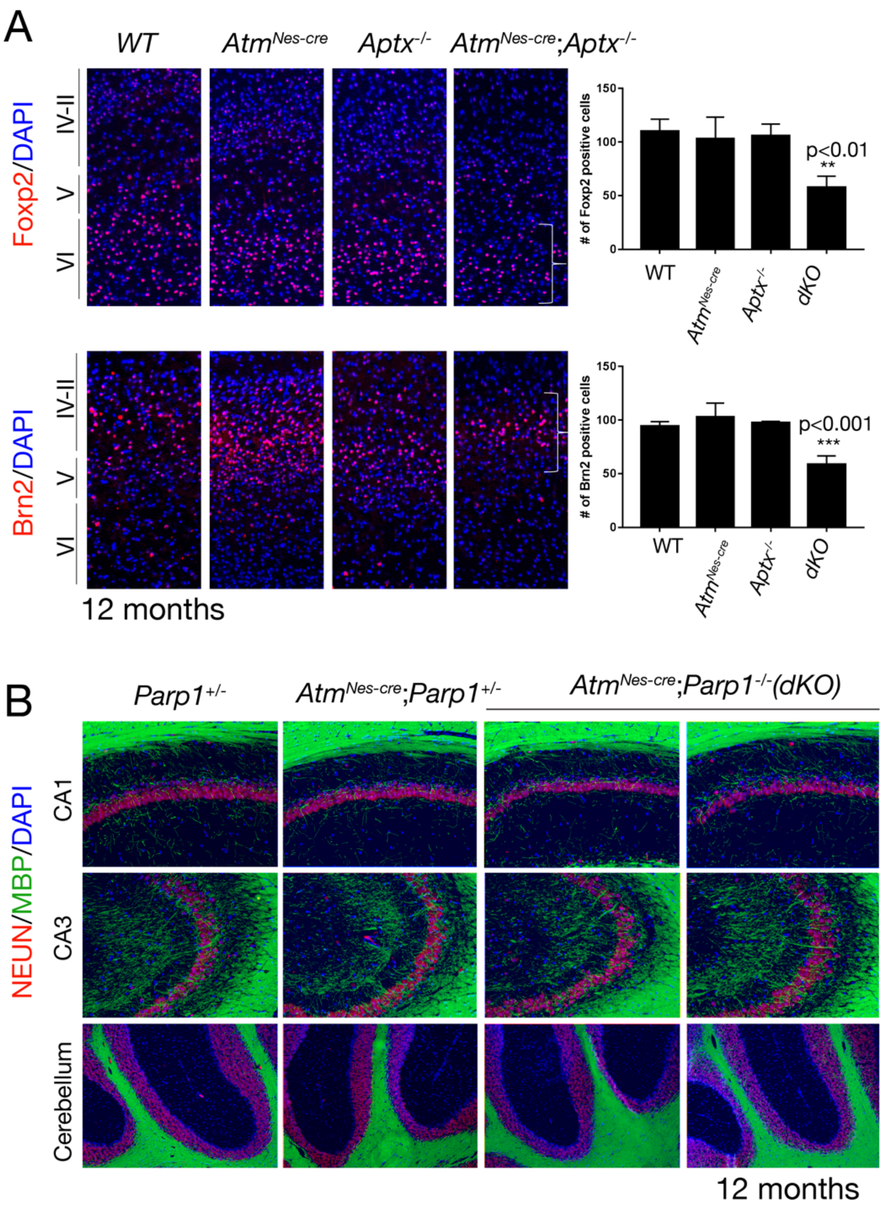

Supplemental Figure 3: Mild decrease in upper layer cortical neurons in double mutant brain. (A) A reduction in the number of upper layer neurons occurs in the *Atm*$^{Nes\text{-}cre}$*;Aptx*$^{-/-}$ 12-month-old double mutant brain. Reduced Foxp2 and Brn2 expressing neurons which reside in layers VI and IV-II respectively (indicated by brackets). (B) Myelin distribution from oligodendrocytes does not show any overt perturbations at 12 months as indicated using Myelin Basic protein. NeuN immunohistochemistry indicates neurons. The following antibodies were used: anti-Foxp2 (1:50, Abnova, PAB12684), anti-Brn2 (1:200, Gene-Tex, GTX114650), anti-Myelin Basic Protein (1:500, Abcam, ab40390) and anti-NeuN (1:500, Chemicon, MAB377).

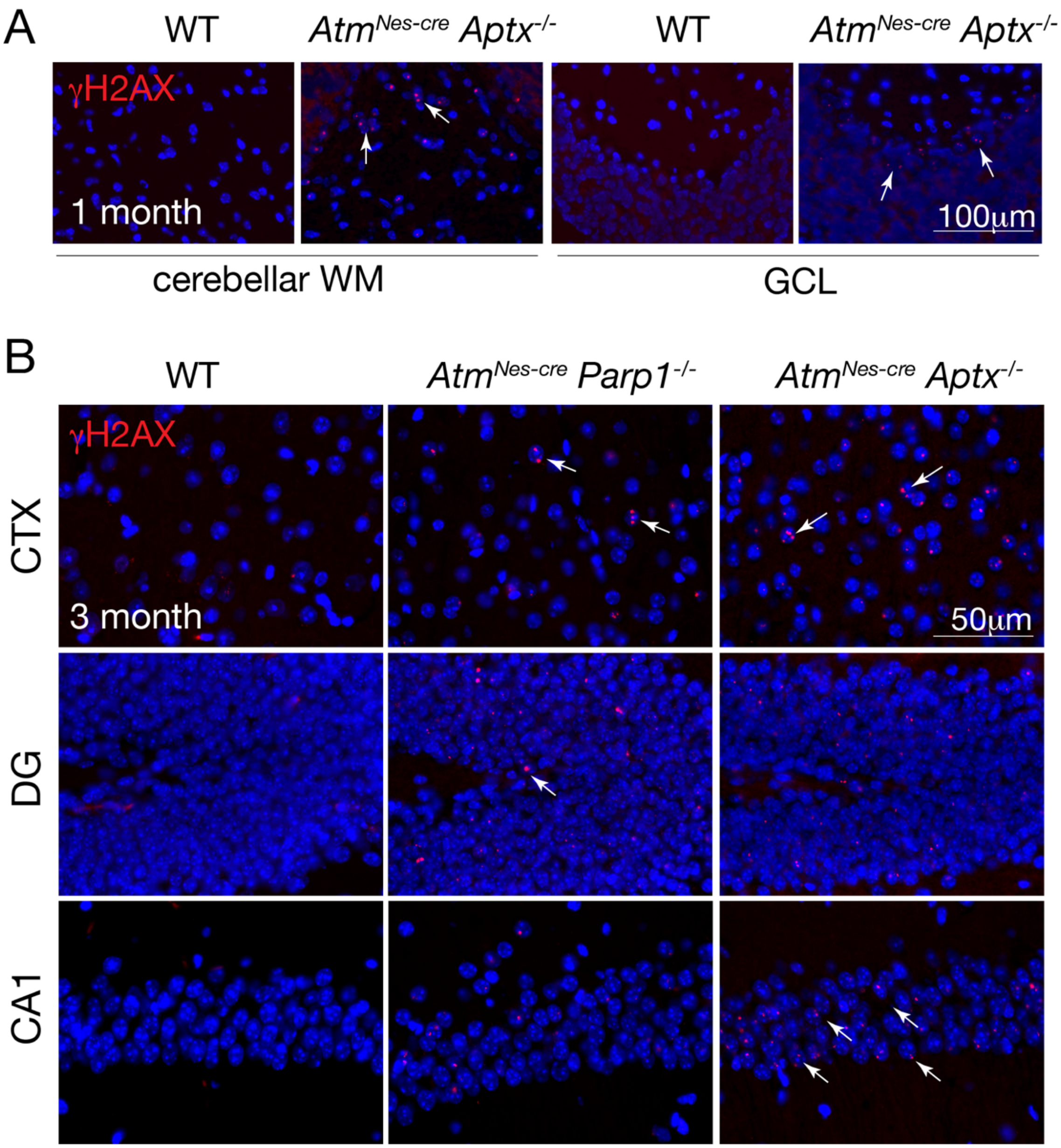

Supplemental Figure 4: DNA damage in the double mutant cerebellum.

DNA damage as indicated by γH2AX nuclear puncta is apparent in the double mutant tissues at 1 and 3 months of age (arrows). Similar levels of DNA damage are seen in the $Atm^{Nes\text{-}cre}$;$Aptx^{-/-}$ and $Atm^{Nes\text{-}cre}$;$Parp1^{-/-}$ tissue. (A) Cerebellar WM is white matter and GCL is the granule cell layer. (B) CTX is cortex, DG is dentate gyrus and CA1 is the Cornu Ammonis region 1. DAPI is the nuclear counter stain.

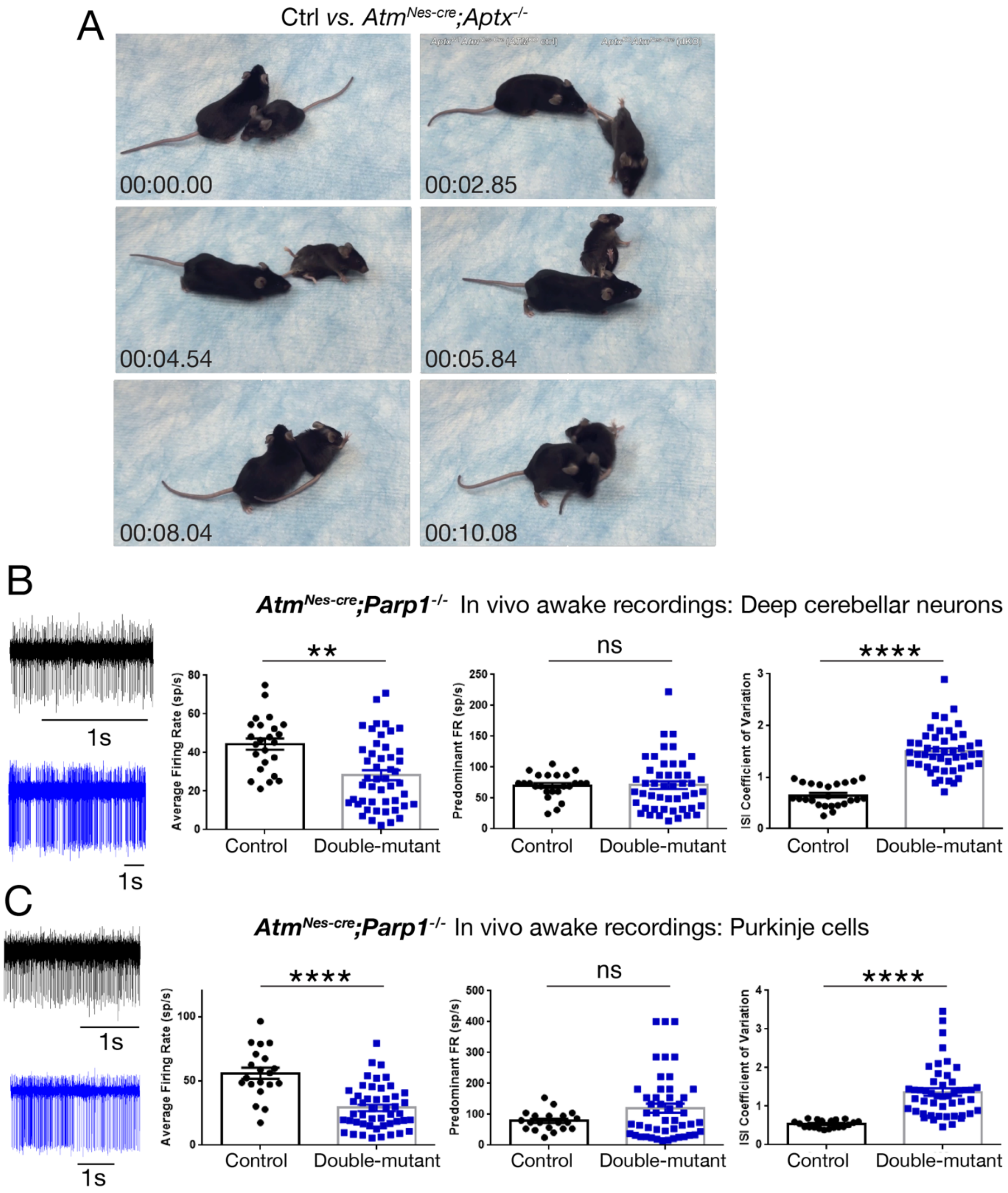

Supplemental Figure 5: Altered electrophysiology in the double mutant cerebellum. (A) A 10 second sequence of still images from a video capture of a 12-month-old ataxic *Atm*$^{Nes\text{-}cre}$*;Aptx*$^{-/-}$ mice showing the profound movement disorders they develop. Numbers indicate the sequential frame captures (in mins: secs.1/100s). Photo credit: Young Don Kwak, SJCRH. (B) Awake head-restrained extracellular recordings from neurons of the deep cerebellar nuclei of *Atm*$^{Nes\text{-}cre}$*;Parp1*$^{-/-}$ double mutant (blue) compared to Control

(black). Quantification of the average firing rate of neurons in the Deep cerebellar nuclei showed a significant decrease in the double mutant compared to the age-matched control mice. To determine if the Purkinje cells are firing irregularly, the coefficient of variation (CV) of the interspike intervals (ISI) was measured. CV ISI is defined as the variability in the timing of spikes within a spike train, therefore changes in the average firing rate of a cell will affect the CV ISI. The ISI coefficient of variation of deep cerebellar nuclei neurons in *Atm*$^{Nes\text{-}cre}$*;Parp1*$^{-/-}$ mice was increased compared with control cerebellum, indicating highly irregular activity (B, C). On the left are example traces recorded from neurons in the deep cerebellar nuclei (B) and Purkinje cells (C) of a control mouse (black) and *Atm*$^{Nes\text{-}cre}$*;Parp1*$^{-/-}$ mice (blue). In both cell types compared to the control mice, there is a decreased average firing rate in *Atm*$^{Nes\text{-}cre}$*;Parp1*$^{-/-}$ mice and increased irregularity of firing as shown by the significant increase in the ISI coefficient of variation. Even though the average predominant firing rate was not increased, some cells showed an obvious increase, indicating burst-firing (ns=non-significant, ****=p<0.0001, **=p<0.01, mean ± S.D.).

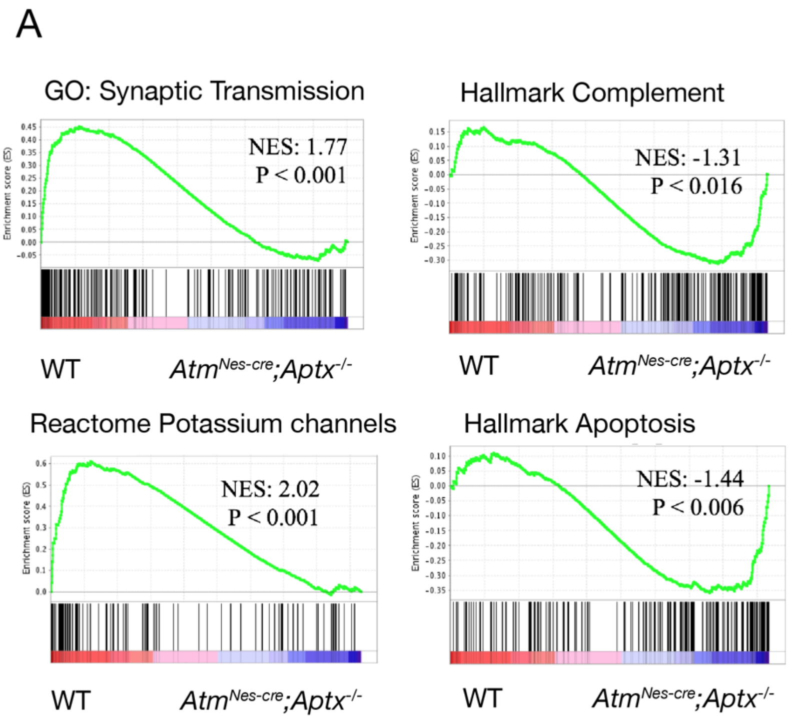

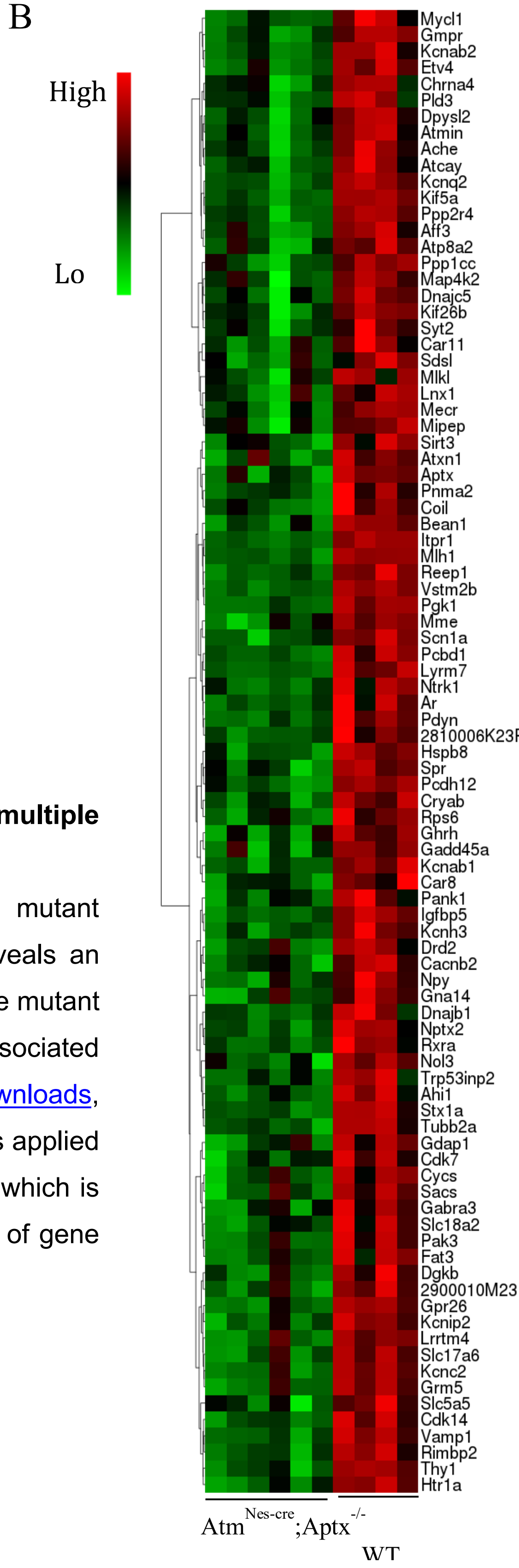

Supplemental Figure 6: RNA-seq analysis identifies multiple defects in important neural homeostasis pathways.

(A) GSEA show altered signaling pathways in the double mutant cerebellum. (B) Text mining from the disease database reveals an extensive list of genes with expression differences in the double mutant cerebellum that are linked to disease states. The disease associated gene list were derived from https://diseases.jensenlab.org/Downloads, and (ref #24). A differential gene expression cutoff of 0.05 was applied to data analysis. This list includes text mining-derived genes which is based on text crawling of abstract and look for co-occurrence of gene with disease names.

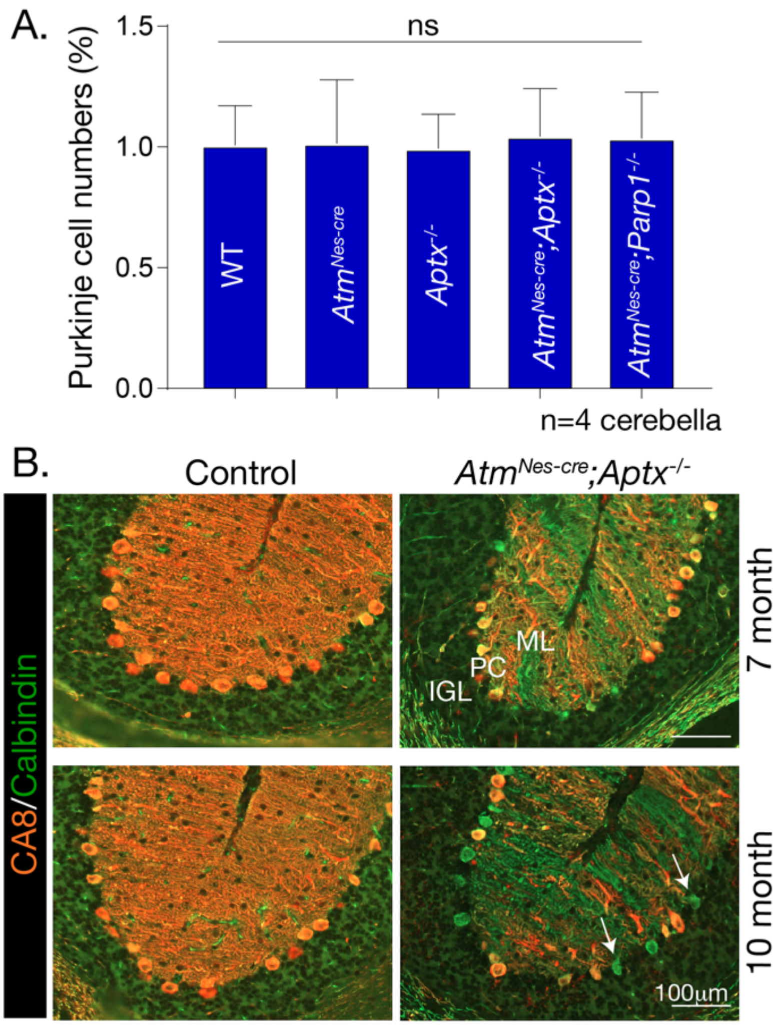

Supplemental Figure 7: (A) Counting of Purkinje cells in various genotypes show no significant reduction in total number of these neurons. Calbindin-positive purkinje cells were counted in matched lobules from all genotypes. Total Purkinje cell numbers were expressed as a fold-change from the WT tissue; the t-test was used to determine significance; ns is not significant. The experiment used 4 individual animals of each indicated genotype. (B) Carbonic anhydrase related gene 8 (CA8, aka CAR8) is also strongly affected in the double mutant cerebellum. Immunohistochemistry of a 7- and 10-month-old *Atm*$^{Nes\text{-}cre}$;*Aptx*$^{-/-}$ cerebellum shows a marked loss of CA8 signal in double mutant Purkinje cells. The Purkinje cell marker, calbindin is used as a second marker; arrows show Purkinje cells that are positive for calbindin that have lost detectable CA8 immunoreactivity, [anti-CA8, 1:500, Novus, NB100-74382]. IGL is inner granule layer, PC is Purkinje cells and ML is the molecular layer.

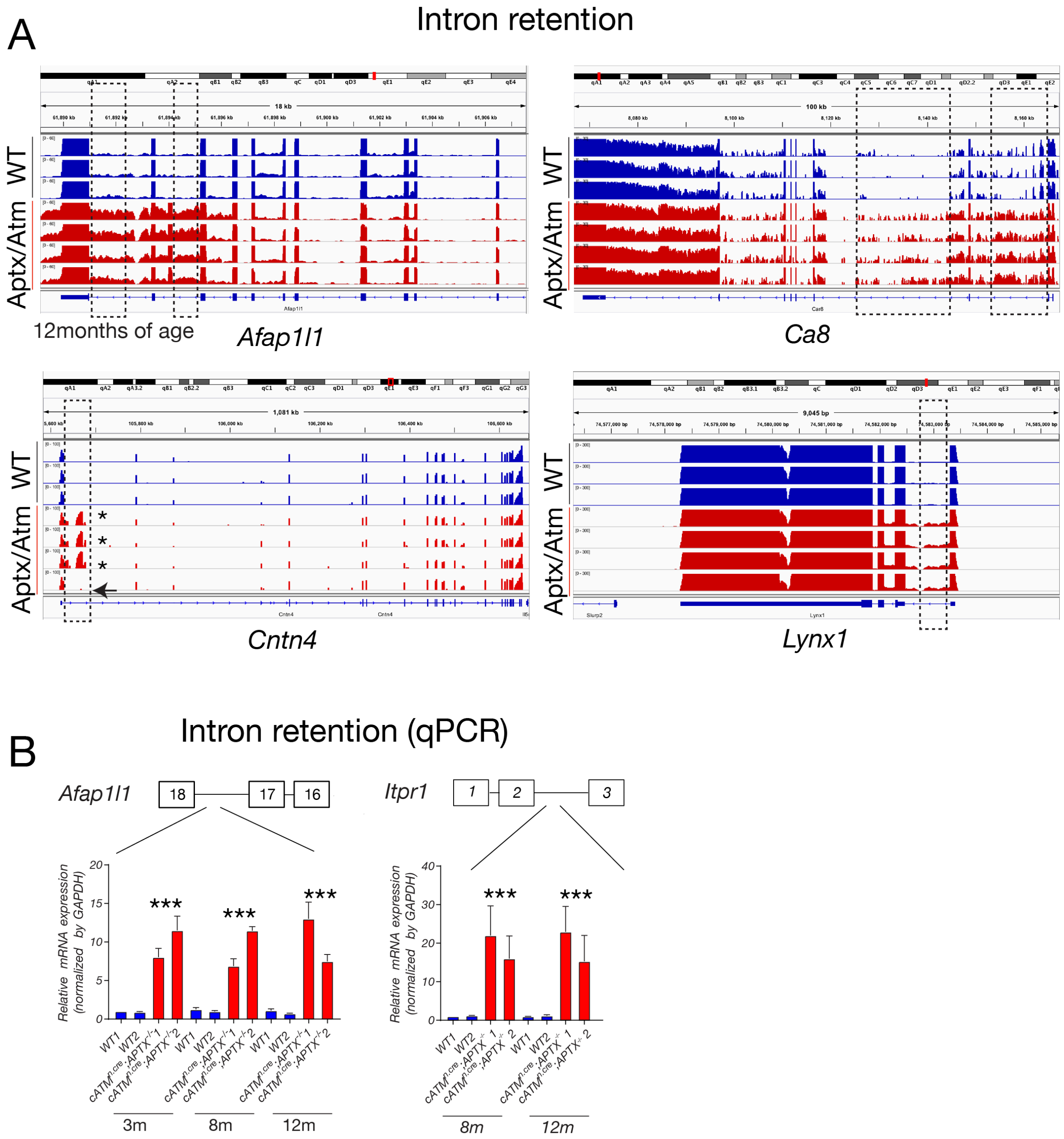

Supplemental Figure 8: Intron retention in the *Aptx*$^{-/-}$*;Atm*$^{Nes\text{-}cre}$ cerebellum. (A) Alignment IVG screen shots of mRNA-seq analysis for *Afap1l1*, *Car8*, *Cntn4* and *Lynx1* shows Intron retention is present in the double mutant samples. Individual mutants usually show the same splicing defects, although in occasional cases the same mutant sample may not exhibit a splice defect in all genes in the cohort of identified common alterations as illustrated for *Cntn4* in one of the double mutants. (B). qPCR of cerebellar cDNA using

PCR primers that recognize retained introns identified by RNA-seq analysis and shows intron retention in the Purkinje cell-specific *Afap1l1* and *Itpr1* message.

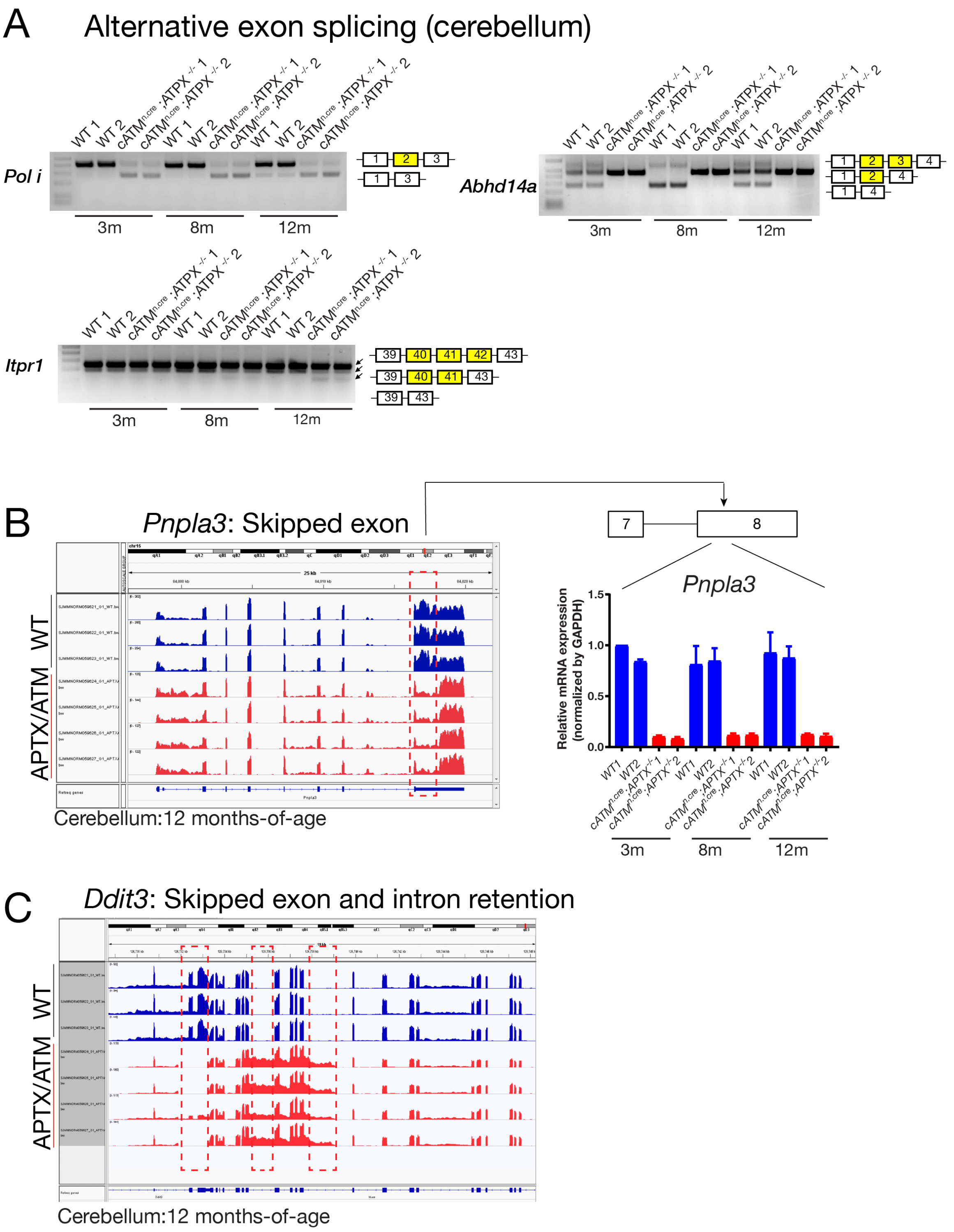

Supplemental Figure 9: Aberrant splicing is a feature of the double mutant mice. (A) qPCR analysis of representative splicing alterations for *Poli*, *Abhd14a* and *Itpr1* involving exon skipping identified by MATS analysis in the $Atm^{Nes\text{-}cre}$;$Aptx^{-/-}$ double mutant

from a range of ages. IGV screenshots of direct RNA-seq analysis in 12-month-old *Atm*$^{Nes\text{-}cre}$;*Aptx*$^{-/-}$ cerebellum shows recurrent skipped exon events for *Pnpla3* (B) and *Ddit3* (C) indicated by red hatched boxes. The adjacent real time PCR expression analysis of *Pnpla3* shows the splicing alterations are present from 3 months-of-age.

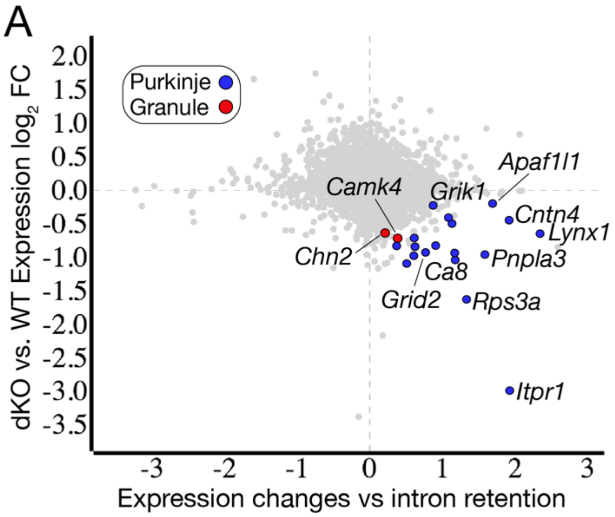

B

Gene	PC expression	GN expression
Ca8	+++++	-
Clec3b	++	-
Cep76	+++++	-
Cntn4	+++	-
Fgf7	+++++	-
Grik1	+++++	-
Itpr1	+++++	-
Kcnip4	++++	-
Lynx1	++++	-
Ndufs5	+++	-
Pnpla3	++++	-
Rps3a	+	-
Sbk1	+++++	-
Tdg	++	-
Them4	++++	-
Ubash3b	++++	-
Camk4	-	++++
Caro2b	-	+++
Chn2	-	+++++

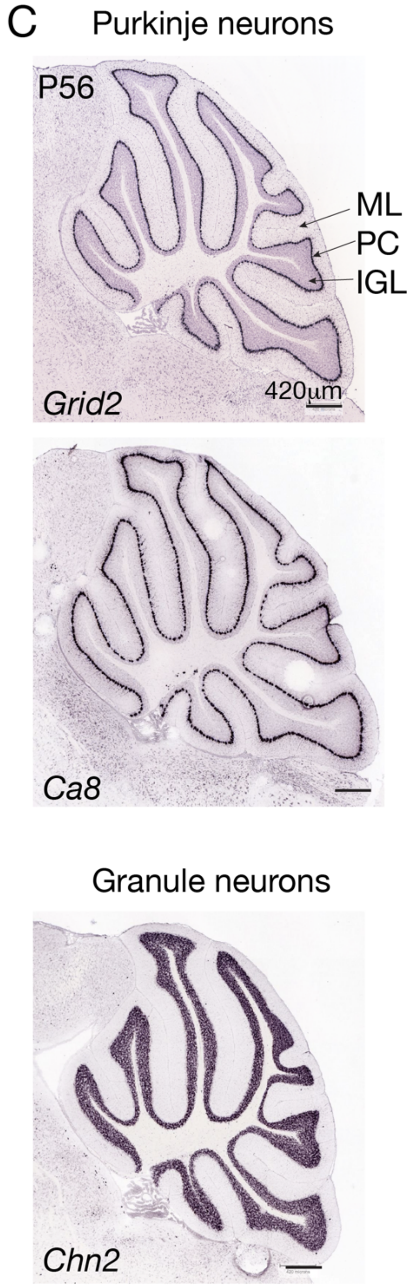

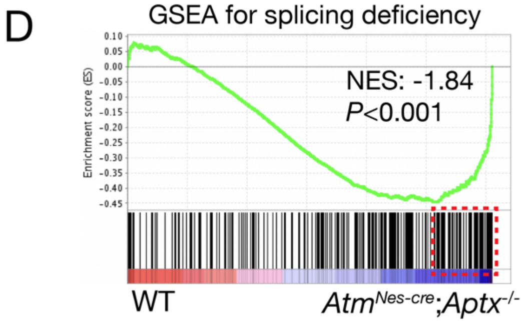

Supplemental Figure 10: Purkinje cells but not Granule neurons show significant retention of introns in the *Aptx*$^{-/-}$*;Atm*$^{Nes\text{-}cre}$ cerebellum. (A) A comparison of cerebellar intron retention measured by the splicing deficiency score compared to gene expression changes identified by mRNA-seq shows a significant correlation (P<0.0001) with decreased expression of Purkinje cell genes that have intron retention. Purkinje specific

genes are listed (blue circles) and granule cell specific genes are shown in red circles. (B) A sample of genes identified in our analysis whose expression in cerebellar cell types was confirmed by in situ analysis at the Allan brain Atlas [http://mouse.brain-map.org/] (C) In situ localization data for the mouse P56 cerebellum was obtained from the Allen Brain Atlas [http://mouse.brain-map.org/]. Image credit: Allen Institute. (D). GSEA score for splicing deficiency in the cerebellum. Genes in the gene-set are down-regulated in APTX ATM. The red boxed area represents the leading-edge splicing deficiency gene set with down-regulated expression. Most genes are Purkinje-expressed genes (Purkinje cell genes listed in *Rosenberg et. al.,* ref #35, are highlighted in blue): Dmtf1, Il15, Accn2, Bcar1, Ogfrl1, Mmp17, Erp29, Kcnc3, Baiap2, Tmem106c, Napa, Slc5a1, Pqlc1, Cdh22, Pxn, Cacna1g, Ptp4a3, Flt3, Rwdd1, 1110008F13Rik, Mpp3, Ppard, Kcnk12, Inpp5a, Dagla, Gas7, Ccdc85c, Ttc9b, Unc13d, Icmt, Cyp11a1, BC042782, Dlg3, Cep76, Dner, AK129341, Tmem40, Zfpm1, Ubash3b, Col18a1, Phtf1, Ipcef1, Stil, Anks1b, Rnf38, Chn2, Trappc5, Loxhd1, Amica1, Il17re, Tmem25, Abi3bp, Hmgb1, Gng13, Plxdc1, Camk4, Dclre1c, Cep72, Rpl3, Tdgf1, Slc22a1, Grid2, Spns3, Mcc, 1700011I03Rik, Mpzl3, Sbk1, Impg2, Gm7819, Gm628, Wdfy1, Dctd, Rps3a, Ca8, Itpr1, Pnpla3, Lynx1.

A

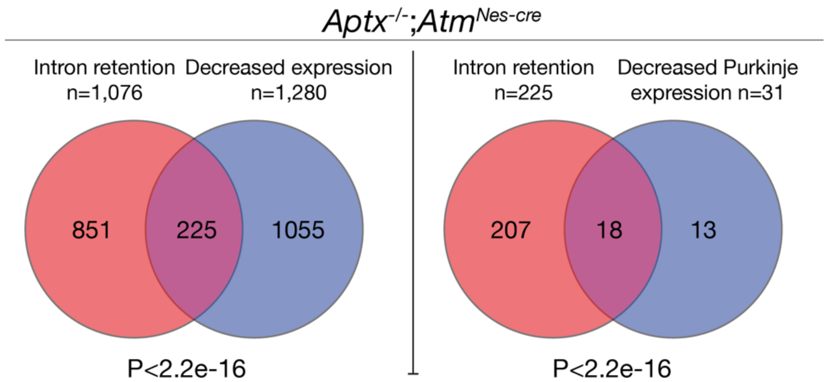

B

Purkinje-specific genes with intron retention
1. *Ankrd33b*
2. *Atp2a3*
3. *Calb1*
4. *Ca8*
5. *Cep76*
6. *Col18a1*
7. *Grid2*
8. *Grid2ip*
9. *Inpp5a*
10. *Itpr1*
11. *Lynx1*
12. *Plcb3*
13. *Plxdc1*
14. *Slc1a6*
15. *Stac*
16. *Tspan11*
17. *Ttll5*
18. *Zfp385c*

C

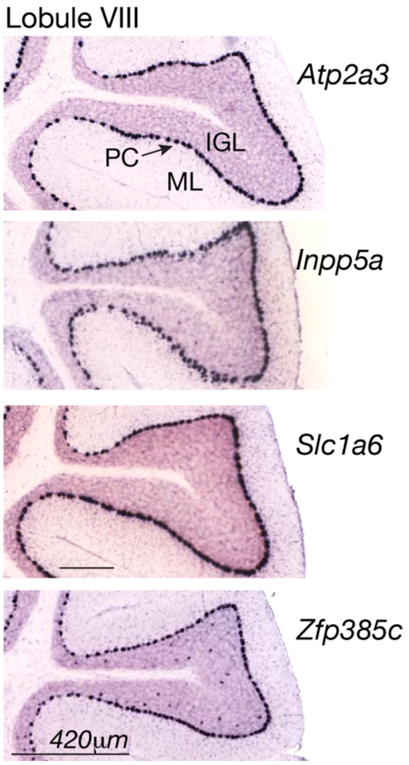

Supplemental Figure 11: Intron retention associated with decreased gene expression occurs in the double mutant cerebellum. (A) Venn diagrams showing the significant increase (p<2.2e-16) in intron retention in genes with decreased expression in the cerebellum (n=225) and those genes listed in the Purkinje cell expression set (Rosenberg et. al., ref #35), n=18, p<2.2e-16. (B) Listing of the genes (n=18) from the Rosenberg et. al., Purkinje cell gene expression set (total PC gene set is n=35). (C) Representative *in situ* expression analysis of a subset of the Purkinje cell genes from *Rosenberg et al* listed in (B). In situ localization in the P56 mouse cerebellum is data obtained from the Allen Brain Atlas [http://mouse.brain-map.org/]. Image credit: Allen Institute.

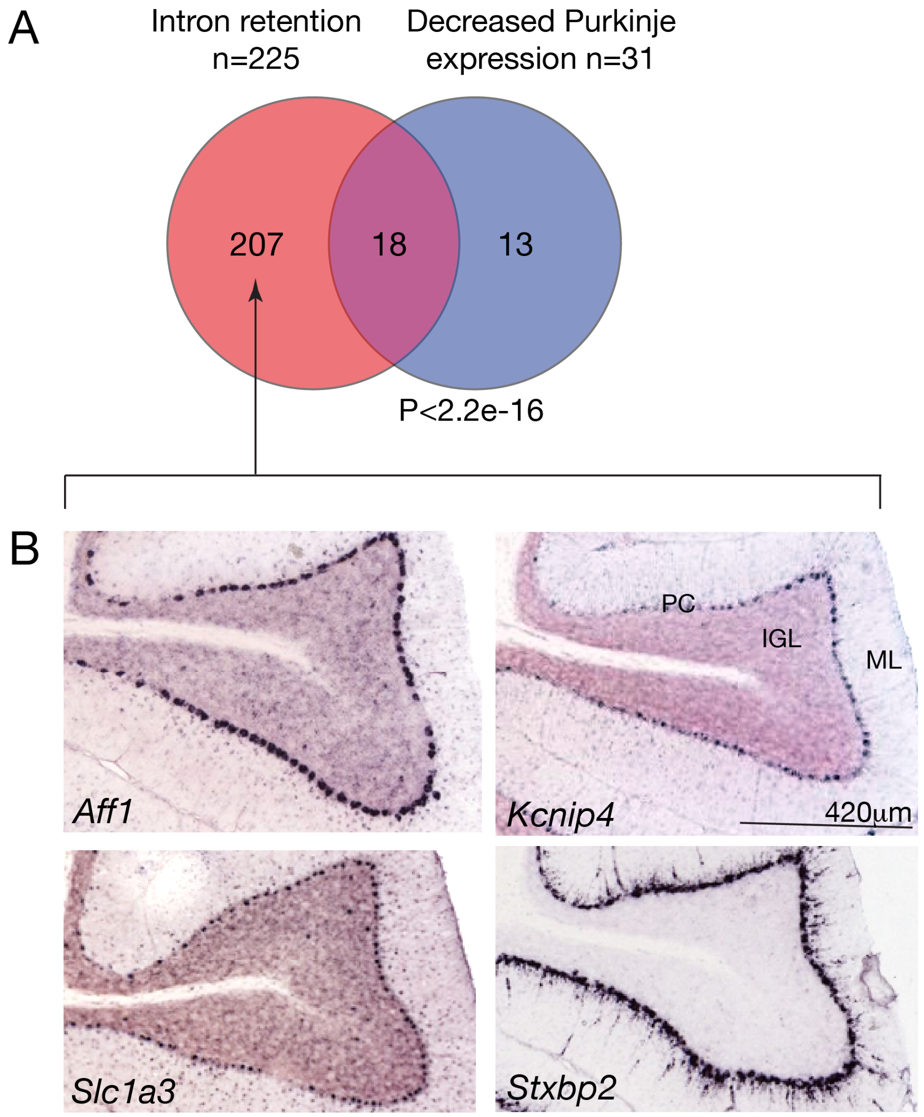

Supplemental Figure 12: Intron retention of Purkinje cell genes in the double mutant cerebellum. (A) Many of the 225 genes showing intron retention and decreased expression are Purkinje cell specific genes that are not listed in established Purkinje gene set lists. (B) analysis of select genes that are decreased in expression in the double mutant cerebellum that show intron retention (via the Splicing deficiency score) are highly/exclusively expressed in Purkinje cells in the cerebellum. In situ localization in the P56 cerebellum is data obtained from the Allen Brain Atlas [http://mouse.brain-map.org/]. Image credit: Allen Institute. This indicates that many of the genes showing intron retention and decreased expression in the double mutant cerebellum are Purkinje cell genes.

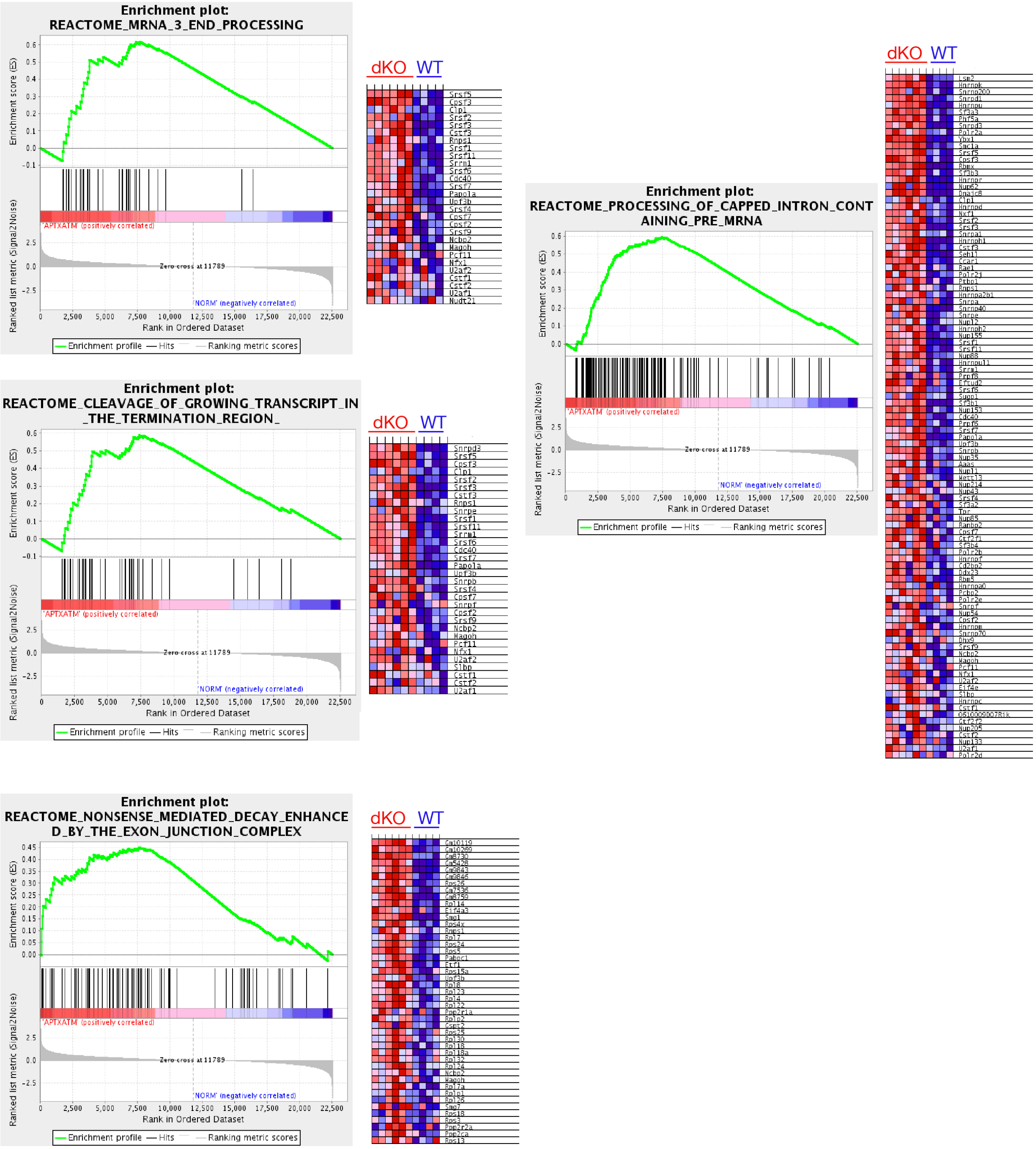

Supplemental Figure 13: GSEA of the mRNA nonsense mediated decay pathway in double mutant cerebellum. Gene set enrichment analysis (GSEA) of the mRNA nonsense-mediated decay pathways shows high significance of this pathway in the double mutant cerebellum based on the related gene sets linked to nonsense mediated decay.

These include, NMD pathways involving 3' end-processing, processing of capped intron containing pre-mRNA, cleavage of the growing transcript in the termination region and NMD enhanced by the exon junction complex. Heat maps of genes involved in the GSEA are shown adjacent to each individual analysis. These data indicate the NMD pathway is active in the double mutant cerebellum.

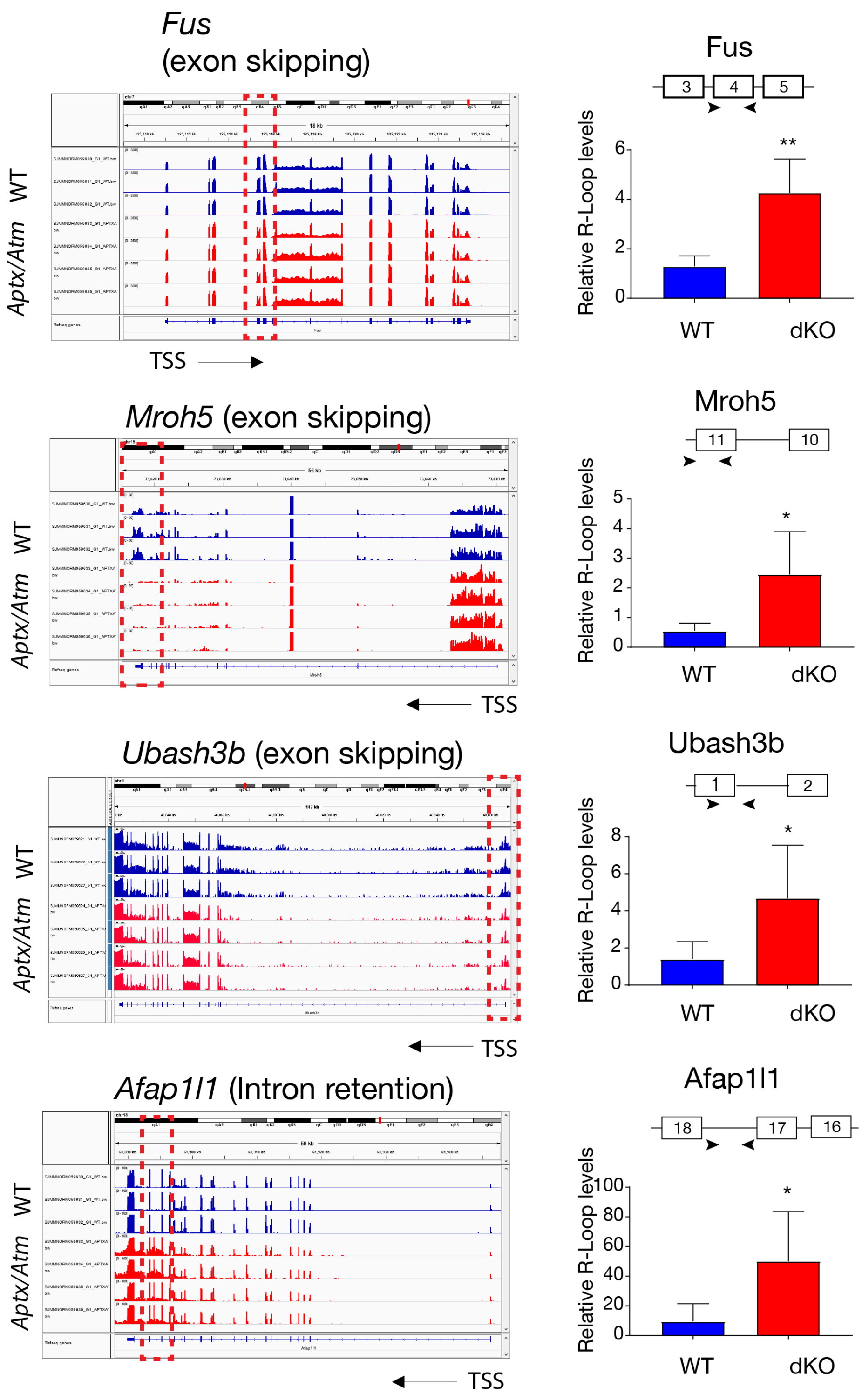

Supplemental Figure 14: Examples of splicing alterations associated with R-loop formation found in double mutant cerebellum. While intron retention was an abnormality present in message from the double mutant cerebellum, other splice defects

were also commonly observed. These splicing alterations were also associated with R-Loop formation in the double mutant. Examples are shown for *Fu*s, *Mroh5*, *Ubash3*, that show exon skipping compared with a gene characterized by intron retention, Afap1l1. In all cases, R-loops are elevated at these loci in the double mutant cerebellum. IGV screen captures show the splicing abnormalities at indicated loci. Adjacent graphs show R-loop quantitation, after normalization with input, at the respective loci using DRIP-qPCR following immunoprecipitation with the S9.6 antibody from 12-month-old WT and dKO cerebellum. Primer locations (arrows) are indicated together with exon/intron arrangement at each gene locus (statistics done using a t-test in GraphPad Prizm; * is $p<0.05$ and ** is $p<0.001$).

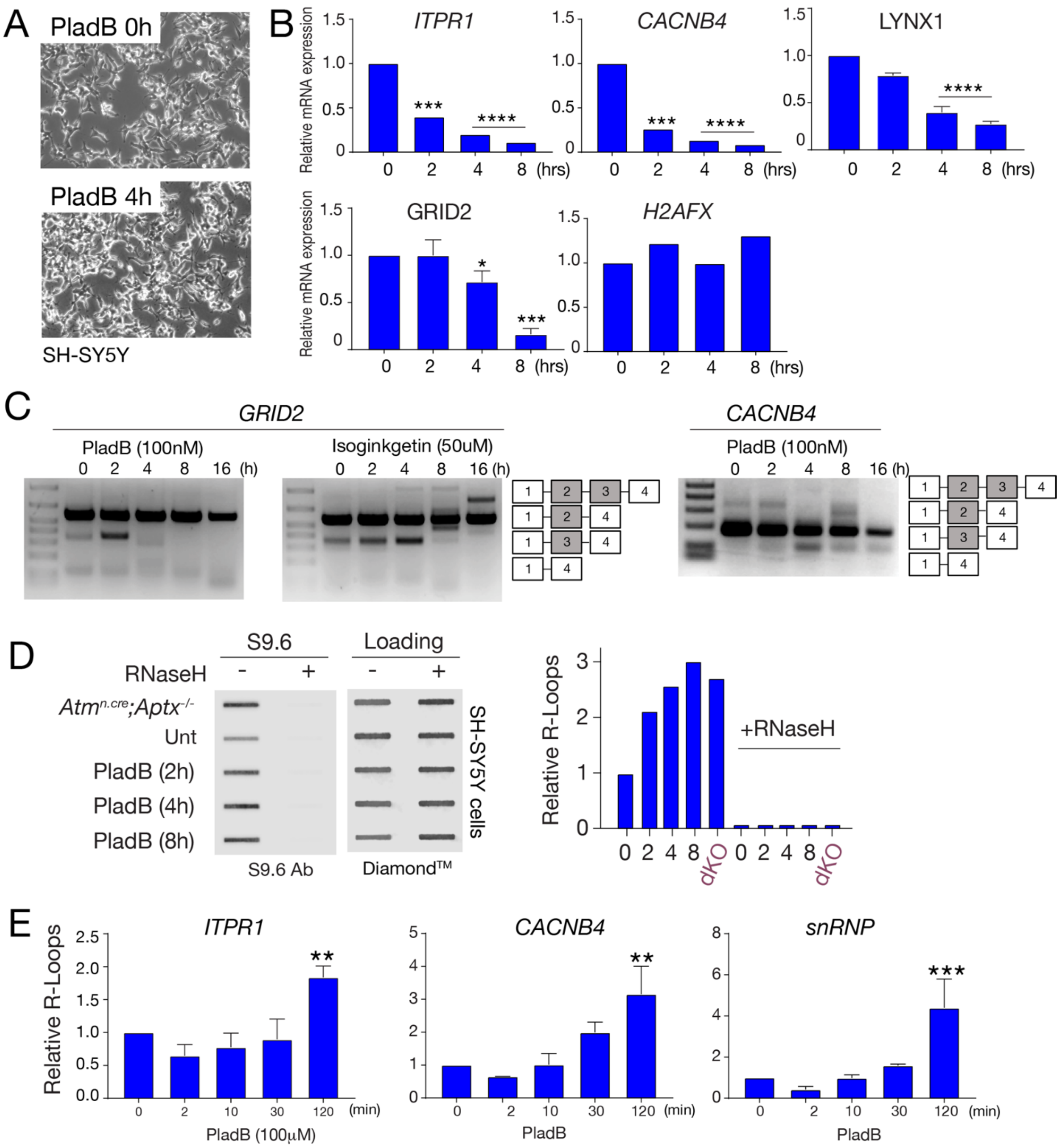

Supplemental Figure 15: Aberrant splicing affects gene expression and causes R-loop formation. (A) Pladienolide B (PladB) treatment of SHSY5Y cells has little effect on viability or morphology. (B) PladB treatment (100nM) causes a decrease in expression of *ITPR1*, *CACNB4*, *LYNX1* and *GRID2*, while *H2AFX*, which doesn't contain introns, is unaffected. (C) PladB or isoginkgetin treatment alters normal splicing of cerebellar genes *GRID2* and *CACNB4*. Numbered boxes adjacent to gels represent relevant exon structure of the regions of PCR amplification. (D) R-loops are increased after PladB (100nM) treatment as identified by immunoblotting with the RNA-DNA S9.6 antibody. RNaseH

effectively abrogates R-loops. Genomic DNA loading was determined using Diamond™ Nucleic Acid Dye. (E) Quantitative PCR of DNA-RNA immunoprecipitation detects R-loops in cerebellum-relevant genes: *ITPR1*, *CACNB4* and *snRNP* (a positive control for R-loop formation). (Statistical analysis was done using a t-test in GraphPad Prizm; * is $p<0.05$ and ** is $p<0.001$ and *** is $p<0.0001$).

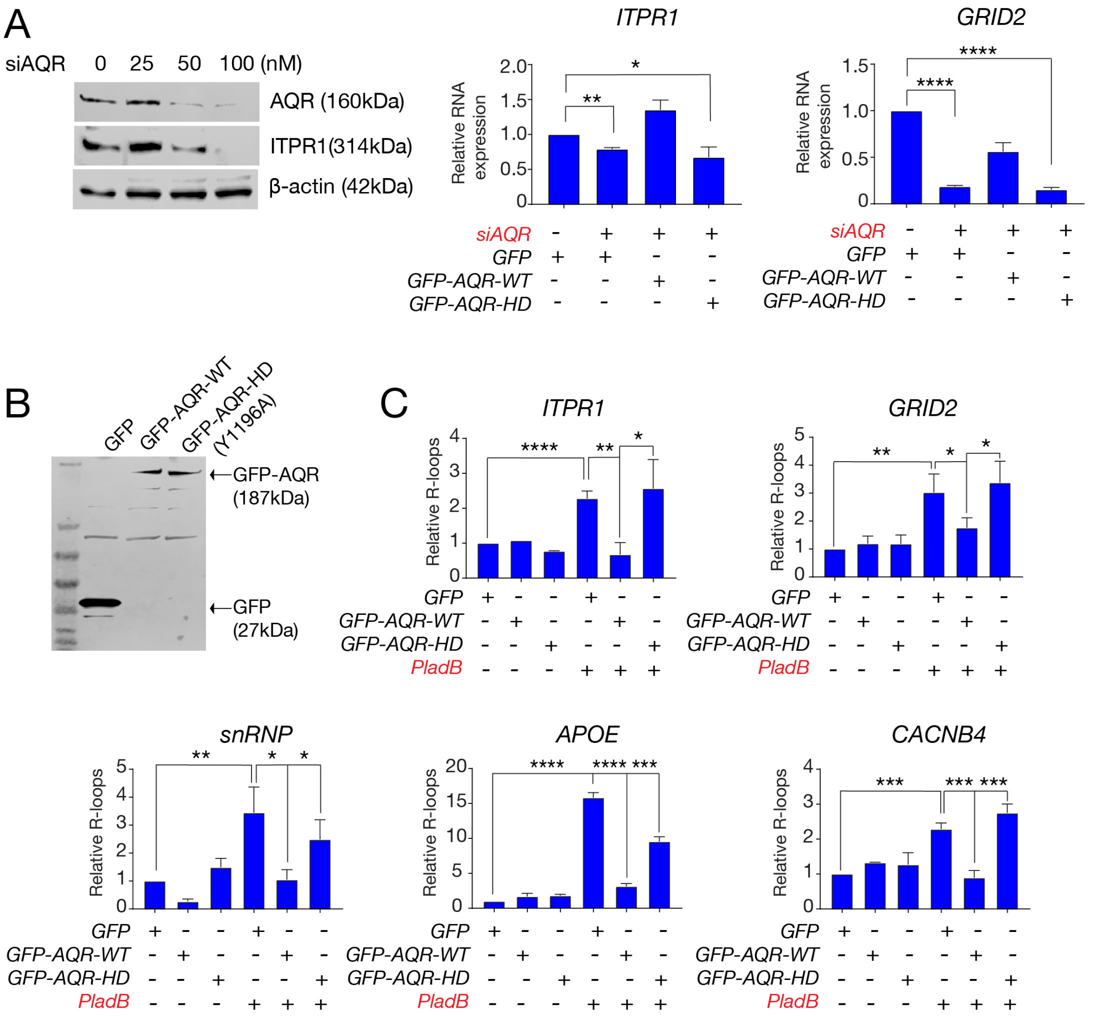

Supplemental Figure 16: The DNA/RNA helicase Aquarius suppresses R-loops. (A). siRNA knockdown of Aquarius (AQR) in SH-SY5Y cells causes a loss of ITPR1. Cells were transfected with siRNA by RNAiMAX (Invitrogen) following the manufacturer's protocol. Expression of an AQR siRNA causes a decrease in *ITPR1* and *GRID2* transcript levels, which are rescued with WT AQR, but not with a helicase-dead (HD) mutation (Y1196A). Expression values were determined by normalization with input. (B) Western blot analysis shows that the GFP-WT and HD mutations of AQR are expressed at equal levels. At 48 h post transfection, cells were treated with spliceosome inhibitors such as PladB for western blotting or RT-qPCR, and DRIP-qPCR. siRNAs used in this study were as follows: *AQR* (Ambion, s18725), and control siRNA (Ambion, Silencer Negative Control #1 siRNA). For Aquarius add-back experiments, *AQR* cDNA was cloned into the pEGFP-C1 vector. Cells were transfected with Aquarius expression plasmid fused with GFP or GFP alone using Lipofectamine 2000 (Gibco BRL, Carlsbad, USA), and then transfected

with siRNA using RNAiMAX 24 h after plasmid transfection. Transfected cells were analyzed for γH2AX foci, ITPR1 expression and R-loop formation. (C) Splicing disruption in SH-SY5Y cells by PladB (100nM) causes R-loop formation, determined by S9.6 DRIP-PCR. WT AQR expression, but not AQR-HD abrogates R-loops, as shown in range of cerebellar-relevant genes. Western blots were done using anti-AQR (IBP160) antibody (rabbit, 1:1000, Abcam cat# ab205303) and anti-GFP (rabbit, 1:3000, Life technology, cat# A11122) followed by horseradish peroxidase-conjugated secondary antibodies (1:5,000, GE Healthcare) and detected using WesternSure® PREMIUM chemiluminescence substrate (LI-COR) and a LI-COR Odyssey Fc (LI-COR). Significance was calculated using a t-test in GraphPad Prizm; *; $P<0.01$, **; $P<0.001$ ***; $P<0.0001$ ****; P<0.00001.

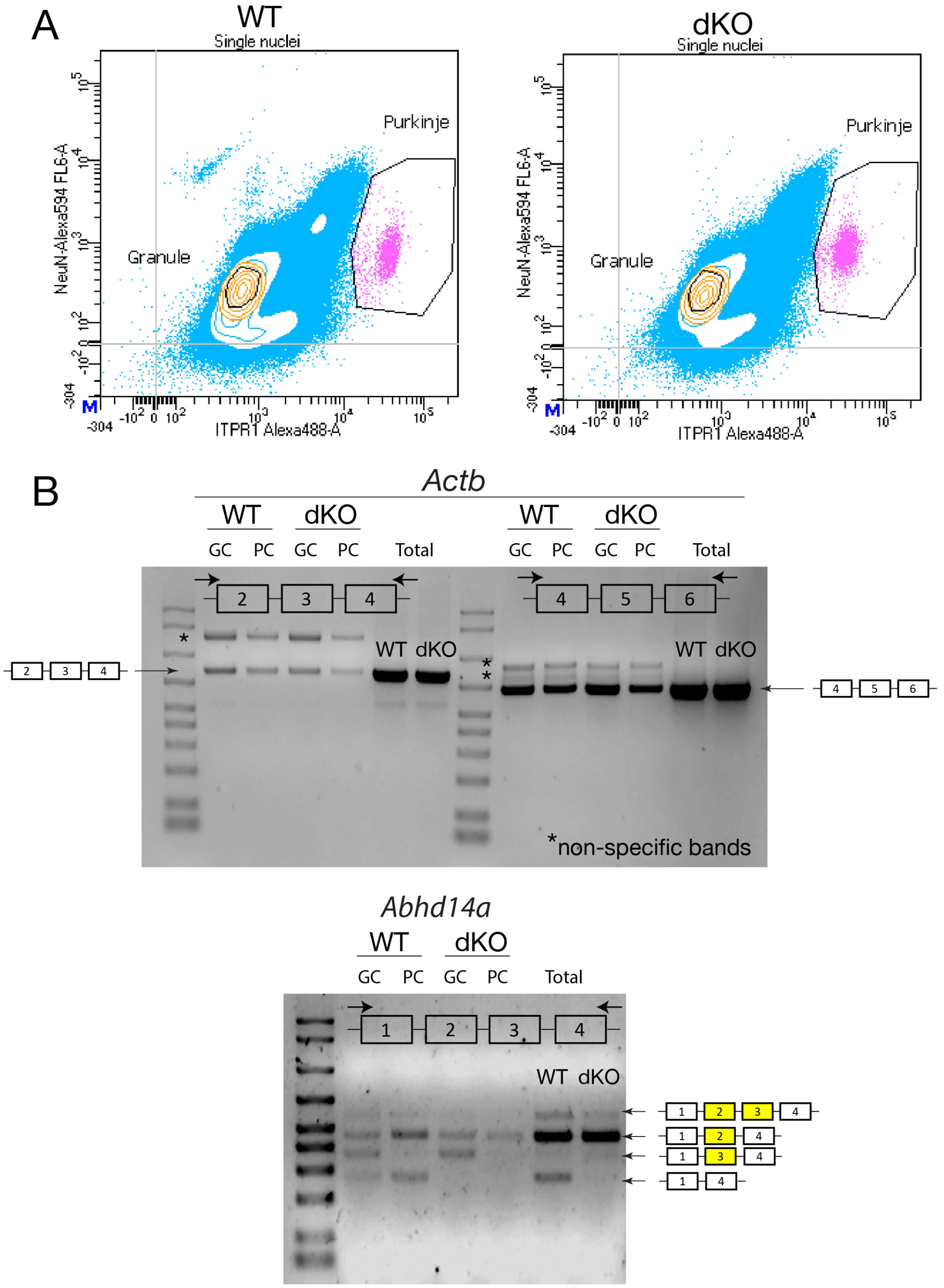

Supplemental Figure 17: Splicing analysis in isolated Purkinje and granule neurons. (A) Cerebella nuclei were isolated via flow cytometry using Purkinje and granule neuron specific antibodies. The flow summary figure indicates the effective identification and separation of each cell type. (B) PCR analysis of cDNA from each cell type indicates *Actb* (actin) does not show differences in splicing in either granule cells (GC) or Purkinje

cells (PC) from the double mutant, while *Adhd14a* shows splicing differences in Purkinje cells from the double mutant. The exon and intron organization over the relevant gene regions are shown together with the primer location used for the PCR, as are the exon splice arrangements in each PCR product for *Actb* and the four different spliced products for *Adhd14a*.

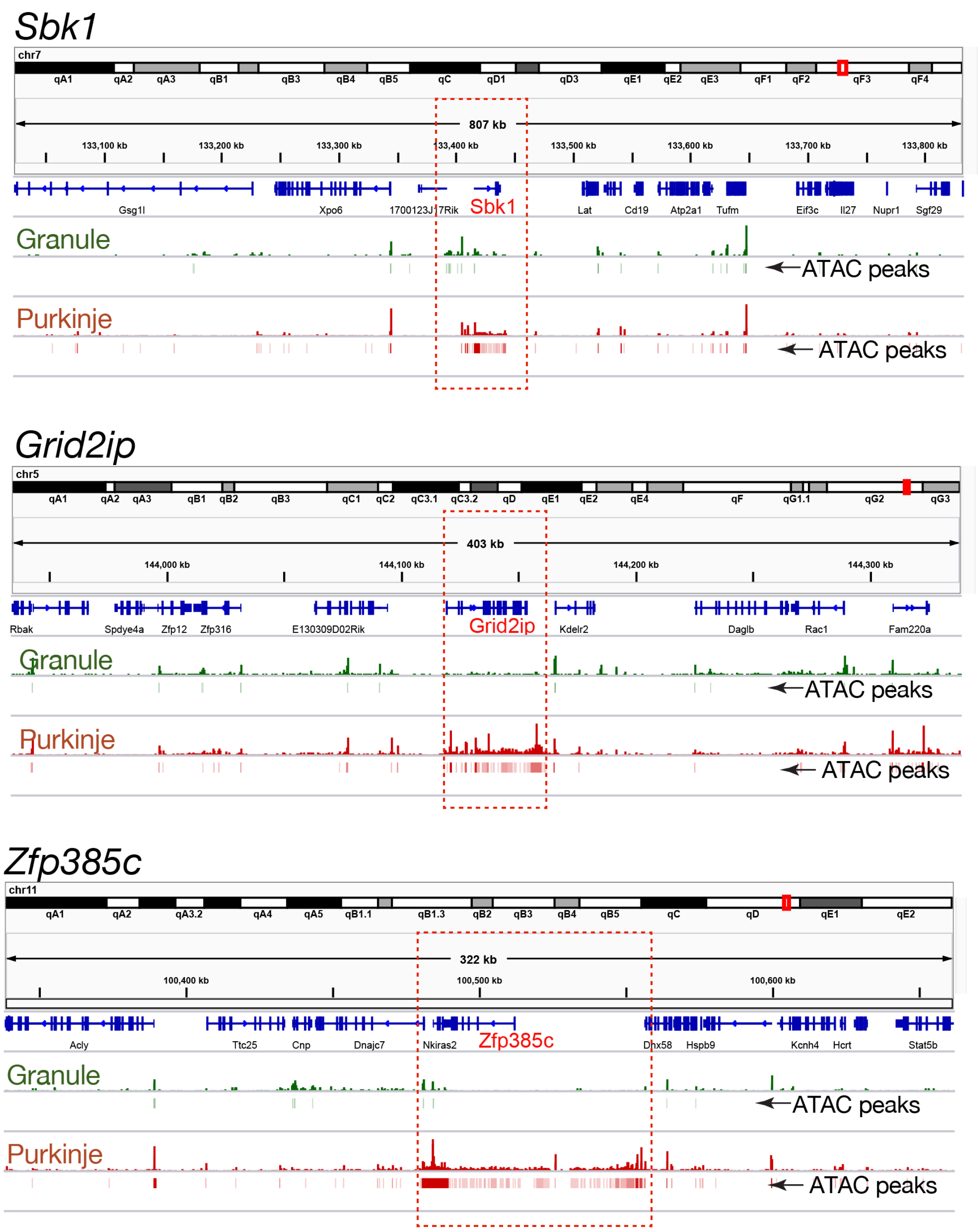

Supplemental Figure 18: Example ATAC profiles at affected genes loci. Representative IGV images of the distribution of ATAC peaks at loci in granule vs. Purkinje neurons showing the unique configuration of the locus in Purkinje cells. Comparative whole gene views of bigwig files and associated bed files of MACS2 peaks for Granule and Purkinje cells revealing the unique openness across the entire gene body of *Skb1*, *Grid2ip* and *Zfp385c* in PCs.

A

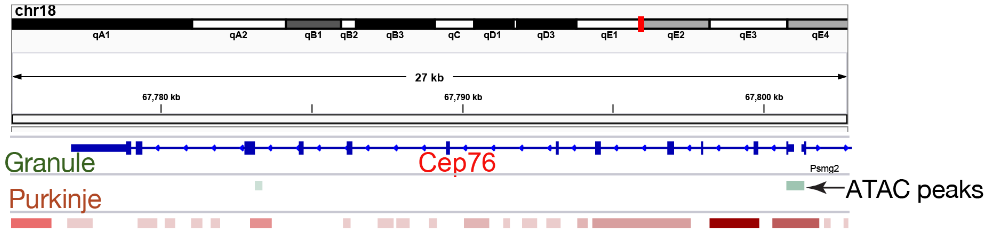

Gene	Peak coverage (kb)	Genomic length (kb)	PC_BP/Genomic ratio[1]	GC_BP/Genomic ratio[2]	Difference
Purkinje cells					
Cep76	19K	24K	0.8	0.08	0.72
Ca8	108K	95K	1.1	0.008	1.1
Grid2	342K	1,448K	0.23	0.004	0.23
Inpp5a	94K	190K	0.49	0.01	0.47
Itpr1	138K	338K	0.41	0.009	0.4
Granule cells					
Camk4	2.0K	256K	0.006	0.018	0.01
Chn2	0.8K	262K	0.002	0.03	0.03
Kctd8	1.3K	232K	0.006	0.03	0.03
Lin7a	1.0K	150K	0.006	0.04	0.04
Zfp521	2.8K	285K	0.001	0.06	0.05

B

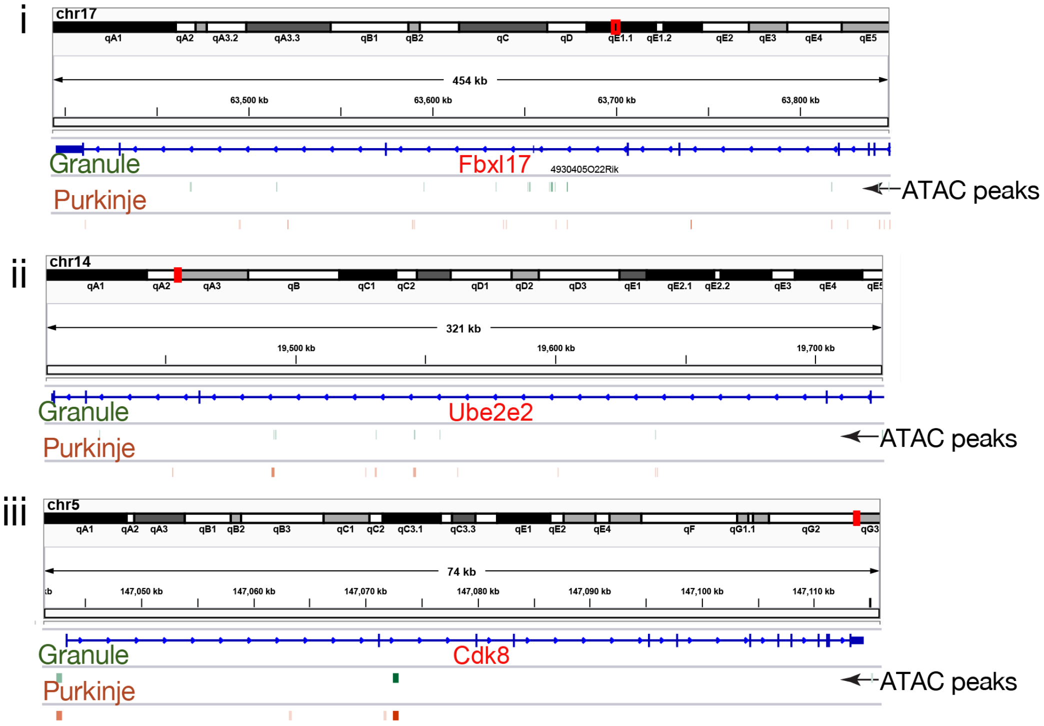

Supplemental Figure 19: ATAC-seq analysis of loci of affected and unaffected genes in the double mutant cerebellum. (A). *Cep76* illustrates the ATAC peak profile of a locus that is affected in Purkinje neurons in the double mutant cerebellum. Summary

of representative difference ratios comparing peak coverage vs. genome length at specific loci in Purkinje and granule neurons. Difference ratios for genes that are affected by genome damage are 10x fold higher in PCs. (B). Examples of ATAC peak patterns at loci that show no expression differences between PCs and GCs in the double mutant cerebellum.

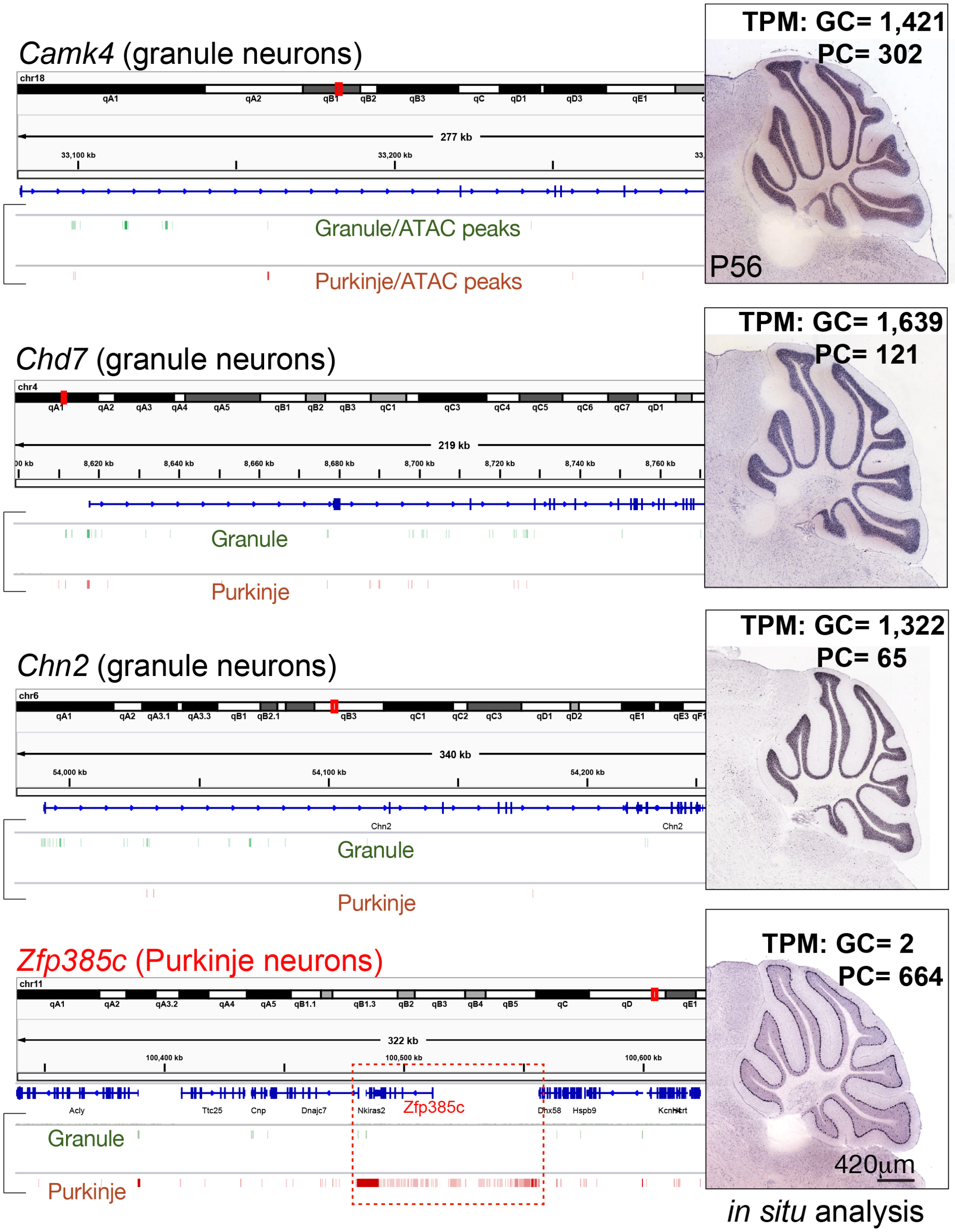

Supplemental Figure 20: ATAC-seq analysis of highly expressed Granule neuron genes. Comparative ATAC-seq profiles for granule cells or Purkinje cells are shown for three genes expressed at high levels in granule cells; *Camk4*, *Chd7* and *Chn2* and *Zfp385c* a gene expressed at relatively high levels in Purkinje cells. The ATAC-seq profile for the granule cell gene loci although showing openness, is distinct to that seen for the

representative Purkinje cell gene shown. TPM is transcripts per million and reflects the relative expression levels in granule cells (GC) and Purkinje cells (PC) as reported by Rosenberg et. al., (*Science* **360**, 176-182 (2018)). In situ localization in the P56 cerebellum is data obtained from the Allen Brain Atlas [http://mouse.brain-map.org/]. Image credit: Allen Institute.

Supplementary data tables:

Table 1: Overview and bulk RNA-seq data.

Table 2: PolyA+ RNA data and splicing deficiency analysis.

Table 3: Multivariate Analysis of Transcriptional Splicing (rMATS) data sets.

Table 4: ATAC peak counts and overlap of cell specific data with whole cerebellum.

Table 5: ATAC peak coverage presented as total bp covered, number of peaks per gene, and ratio of peak coverage to length per Gene.

Table 6: PCR primer sequences used in this study.
